# In Situ Formation of TiB_2_ in Fe-B System with Titanium Addition and Its Influence on Phase Composition, Sintering Process and Mechanical Properties

**DOI:** 10.3390/ma12244188

**Published:** 2019-12-13

**Authors:** Mateusz Skałoń, Marek Hebda, Benedikt Schrode, Roland Resel, Jan Kazior, Christof Sommitsch

**Affiliations:** 1 IMAT Institute of Materials Science, Joining and Forming, Graz University of Technology, Kopernikusgasse 24/1, 8010 Graz, Austria; mateusz.skalon@tugraz.at; 2 Institute of Materials Engineering, Cracow University of Technology, 24 Warszawska ave, 31-155 Cracow, Poland; mhebda@pk.edu.pl (M.H.); kazior@mech.pk.edu.pl (J.K.); 3 Institute of Solid State Physics, Graz University of Technology, Petersgasse 16/II, 8010 Graz, Austria; b.schrode@tugraz.at (B.S.); Roland.resel@tugraz.at (R.R.)

**Keywords:** titanium diboride, titanium, boron, iron, dilatometry, liquid phase, phase composition, sintering

## Abstract

Interaction of iron and boron at elevated temperatures results in the formation of an E (Fe + Fe_2_B) eutectic phase that plays a great role in enhancing mass transport phenomena during thermal annealing and therefore in the densification of sintered compacts. When cooled down, this phase solidifies as interconnected hard and brittle material consisting of a continuous network of Fe_2_B borides formed at the grain boundaries. To increase ductile behaviour, a change in precipitates’ stoichiometry was investigated by partially replacing iron borides by titanium borides. The powder of elemental titanium was introduced to blend of iron and boron powders in order to induce TiB_2_ in situ formation. Titanium addition influence on microstructure, phase composition, density and mechanical properties was investigated. The observations were supported with thermodynamic calculations. The change in phase composition was analysed by means of dilatometry and X-ray diffraction (XRD) coupled with thermodynamic calculations.

## 1. Introduction

Due to a stable microstructure and mechanical properties under exposure to long-term thermal neutron irradiation, borated stainless steels find extensive applications in the nuclear industry [1,2]. Potential applications of boron steels in nuclear industry are, e.g., control/shutoff rods in nuclear reactors, sensor for neutron counting, shapes for neutron shielding, dry transportation casks, spent fuel rod storage racks. The advantage of borated stainless steel produced by powder metallurgy technology in comparison to the conventionally produced cast/wrought products is that they contain much smaller and more uniformly distributed boride particles [1,2]. Moreover, it is possible to obtain a material whose properties have lesser decrease in ductility and impact toughness as the boron content increases compared with similar boron-containing cast/wrought borated stainless steels. The application of boron in powder metallurgy as addition to ferrous alloys has been of interest to numerous researchers over the last years [3,4,5,6,7,8,9,10,11,12]. The main cause of this interest is the eutectic reaction between iron and boron, resulting in the appearance of a liquid phase which efficiently improves mass transport by intensifying a diffusion process [7]. Even small amounts of boron (0.4–0.6 wt %) added to ferrous powder may result in great densification of sintered compacts reaching almost full relative density [7,9]. In accordance with a series of experiments [9,13,14,15,16], the sintering process is activated through the presence of a liquid phase by acting in three stages: (i) rearrangement (liquid spreading), (ii) solution-reprecipitation of a base material and (iii) microstructural coarsening [9]. Unfortunately, boron is almost non-soluble in an iron-based solid matrix, so it remains at the grain boundaries after the sintering process in the form of hard and brittle borides creating an almost continuous network surrounding the individual grains, therefore significantly influencing the material properties. Controlling the borides’ morphology and/or crystallographic network could considerably broaden the application field of steel parts manufactured by Powder Metallurgy (PM). It was already proved that the addition of titanium to iron-based cast alloys may result in the formation of titanium diboride (TiB_2_) [17,18]. Such an interaction, however, was not yet investigated in sintered particular materials. The objective of the present paper was to investigate the influence of titanium addition on phase changes and sintering behaviour in the Fe-B system. This topic is of high importance due to the possibility of improving the ductility of the particulate borated steels.

## 2. Materials and Methods

Water atomised iron powder of >99% purity delivered by Sigma Aldrich (St. Louis, MO, USA) was used as a base powder. Boron of >99.7% purity (grain size < 1 μm) delivered by Sigma Aldrich was added to the blend in an amount of 0.8 wt % in order to induce a eutectic liquid appearance during the sintering process and consequently the appearance of Fe_2_B borides. The titanium of 99.9% purity delivered by VWR company (Radnor, PA, USA) was also added to the blend as a powder in a stepwise increasing amount in two different grain size classes as listed in Table 1. The varying grain size distribution of titanium allowed for control of the initial reaction surface between titanium and the eutectic liquid. Blends were mixed using a Turbula-type mixer for 12 h in order to assure homogeneity of blends.

The mixed powders were single-axially compacted under a pressure of 600 MPa informs of (a) 4 mm × 4 mm × 20 mm cuboid (dilatometric samples); (b) a 5 mm × 20 mm cylinder (microstructure and density check) and (c) a cuboid 6 mm × 12 mm × 30 mm for Transverse Rupture Strength test (TRS). During the consolidation of the samples, no lubricant was used. Dilatometric tests were carried out in a Netzsch DIL 402C dilatometer (NETZSCH-Gerätebau GmbH, Selb, Germany) under hydrogen atmosphere of 99.9999% purity and a flow rate of 100 mL/min. The heating rate was equal to 20 °C/min, the cooling rate was 10 °C/min while the isothermal temperature and time were 1240 °C and 30 min, respectively. Nabertherm P330 tubular (Nabertherm, Lilienthal, Germany) furnace was used for sintering both the TRS and the cylindrical samples under the same conditions as dilatometric samples. Material porosity was checked using the standard water displacement method. Scanning Electron Microscope (SEM, TESCAN Brno s.r.o., Brno, Czech Republic) TESCAN Mira3-SEM equipped with an Energy Dispersive X-Ray (EDX) spectrometer was used to evaluate the localisation of titanium in the microstructure. Specular X-ray diffraction patterns were collected on a PANalytical Empyrean diffractometer (Malvern Panalytical, Herrenberg, Germany) using a wavelength of 0.154 nm. On the primary side, the machine was equipped with a 1/8° receiving slit, a 10 mm beam mask and a multilayer mirror for monochromatisation and formation of a parallel beam. On the secondary side, an 8 mm anti-scatter slit, 0.02 Rad Soller slits and a PANalytical PIXcel 3D detector (Malvern Panalytical, Herrenberg, Germany) in scanning line mode were used. All thermodynamic calculations were performed using Thermo-Calc software v.3.0 (Thermo-Calc Software, Solna, Sweden) with the TCFE6 database appended. State of full equilibrium was assumed.

## 3. Results and Discussion

### 3.1. Thermodynamic Calculations

**Figure 1:**
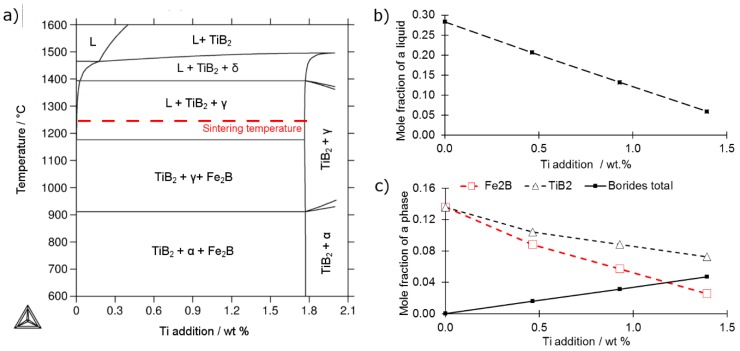
(**a**) Calculated phase diagram of the system (Iron + 0.8B wt %) + Ti, where: α, Ferrite; γ, Austenite; δ, Delta Ferrite; L, Liquid; (**b**) calculated phase fraction of a liquid phase at the sintering temperature of 1240 °C; (**c**) calculated mole fraction of borides as a function of titanium addition at the room temperature.

Figure 1a presents the calculated phase diagram in the Fe-B-Ti system along with the temperature increase for various amounts of titanium addition. The calculations indicate that an increasing amount of titanium results in disappearance of Fe_2_B boride which gets replaced by TiB_2_ (Figure 1c). According to literature, the reason for this replacement is the relatively high difference in Gibbs free energy of formation between the two borides reaching 150 kJ/mol throughout the whole temperature range between 400–1800 °C [18]. Such a replacement should be also accompanied by a decreasing amount of a eutectic liquid at sintering temperature of 1240 °C as presented in Figure 1b.

Figure 1b presents the mole fraction of a liquid phase for the constant sintering temperature of 1240 °C, showing a decreasing amount of liquid phase with an increasing titanium addition. This is connected to changes in borides composition due to the replacement of Fe_2_B by TiB_2_ borides (Figure 1c). As a result of the difference in molar volume (Fe_2_B: 16.76 mol/cm^3^, TiB_2_: 15.37 mol/cm^3^) and the difference in metal-boron stoichiometry (each mole of TiB_2_ contains two times more boron than a mole of Fe_2_B), titanium diboride is capable of binding the same amount of boron in lesser volume as compared to Fe_2_B (Figure 1c). Moreover, due to the high melting point of TiB_2_ it is solid in the sintering temperature; therefore, calculations indicate the decreasing amount of a liquid phase in Figure 1b. For the experimental studies, the compositions of blends (Table 1) were selected based on the presented thermodynamic calculations to assure a considerable but linear decrease of the eutectic liquid amount at the sintering temperature (Figure 1a,b).

### 3.2. Dilatometric Analysis and Density Changes

**Figure 2:**
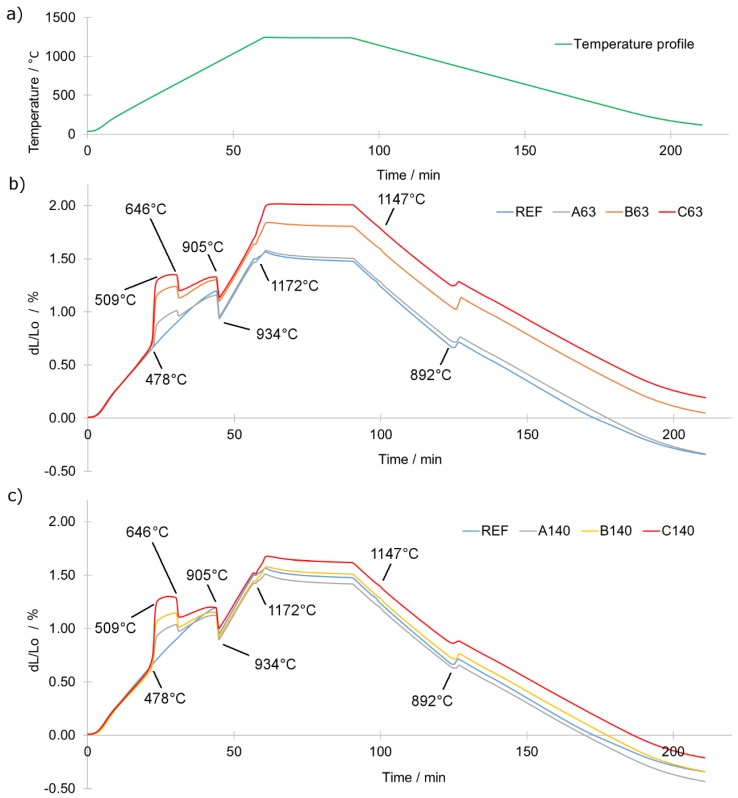
(**a**) Temperature profile of dilatometric tests; (**b**) dilatometric curves of samples A63, B63 and C63; (**c**) dilatometric curves of samples A140, B140 and C140.

To study the influence of the particle size of the additional titanium on the dilatometric behaviour, length changes of the investigated samples were recorded over a broad temperature range (Figure 2a). Figure 2b,c shows the results for the different particle sizes. Quantification of the dilatometric changes was performed by calculating the length changes in specific regions (measurement scheme and detailed values in supplementary). Independently of the titanium particle size, the measurements showed rapid swelling of the sample at the temperature of 478 ± 2.3 °C and ends up at the temperature of 509 ± 1.9 °C. It is then followed by a rapid shrinkage starting at 646 ± 0.3 °C. The former reflects the temporary formation of titanium hydride (TiH_x_) due to interaction of elemental titanium and hydrogen derived from the atmosphere. With an increase of titanium content, the swelling effect was more pronounced. On the other hand, the shrinkage observed at around 646 ± 0.3 °C reflects the intensive release of hydrogen due to thermally induced dehydrogenation, i.e., decomposition of TiH_x_. This decomposition is finished before the temperature of 905 °C is reached because TiH_x_ becomes unstable below this temperature [19,20]. This also means that above 905 °C, titanium is available in elemental form. These observations stay in a good agreement with previous studies [19,20,21]. The swelling of titanium particles originated from its hydrogenation can introduce large tensile stress on neighbouring iron particles and therefore can be a potential source of an additional porosity in the sintered compact. After the dehydrogenation process, the transition of the base iron powder from the crystallographic form of BCC to FCC starts at 905 ± 4.4 °C and ends at 934 ± 0.3 °C (Figure 2b,c).

**Figure 3:**
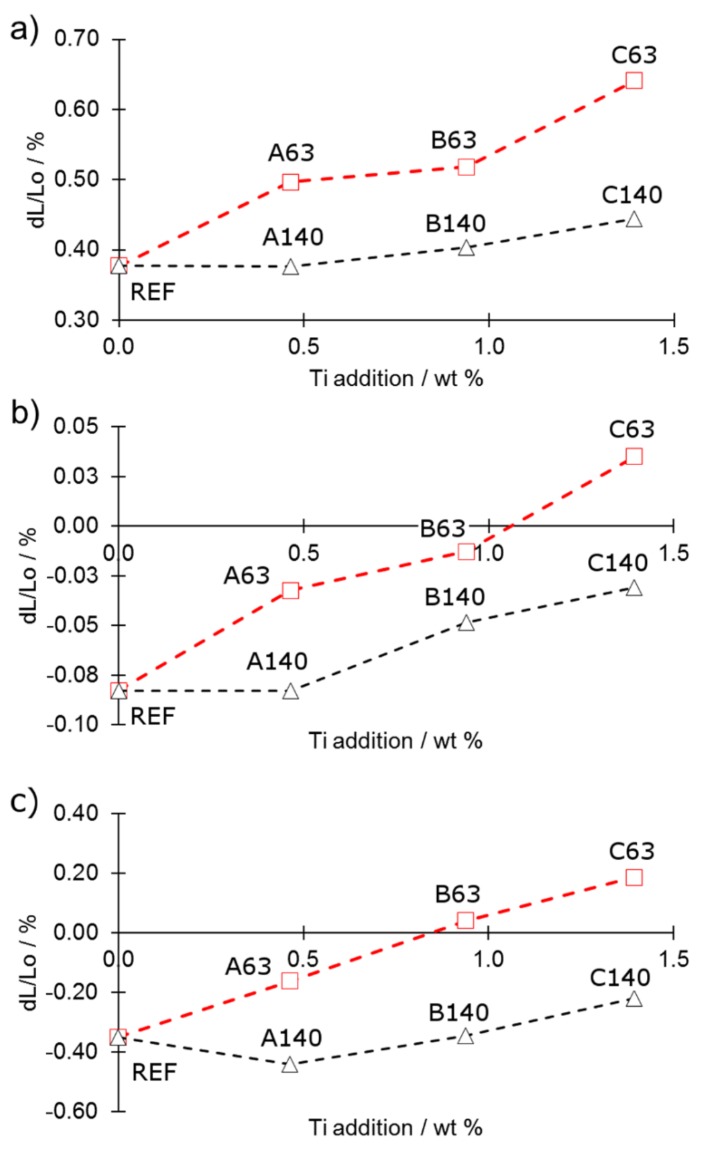
Dilatometric changes: (**a**) in the region between the occurrence of the eutectic phase and isothermal step; (**b**) at an isothermal step and (**c**) total changes.

Consequent heating results in the eutectic reaction between iron and boron starting at a temperature around 1172 °C, which is in good agreement with previous studies [8,10]. The presence of the eutectic liquid enhances mass transport causing densification of the material. As a consequence, shrinking becomes a more dominant effect than thermal expansion. This is observed as a length reduction of the sample in the dilatometric curves. This effect is presented in Figure 3a,b in detail. As can be easily seen in Figure 3a,b the more titanium was introduced the higher was swelling at temperatures of eutectic liquid occurrence. This shows that the more titanium was added the less effectively mass transport occurred at a temperature above 1172 °C. Moreover, the total dimensional changes (Figure 3c) show the same trend. The dilatometric results suggest that fine titanium particles reacted more effectively with the eutectic liquid rather than coarse ones.

Such an observation indicates a decreasing amount of the liquid phase along with the increasing addition of the titanium. The smaller was the particle size of titanium the stronger was its influence dilatometric behaviour. As presented in Figure 3c, use of high additions of titanium of fine particles (C63) may lead even to swelling in respect to the compacted sample. This may affect negatively the final relative density of the sintered compacts. The cooling part of dilatometric curves (Figure 2a–c) shows no evidence of elemental titanium presence as it was observed in the heating stage. The only visible effects are a solidification of borides at 1147 ± 2.1 °C and transformation of austenite back to ferrite starting at 893 ± 2.4 °C.

**Figure 4:**
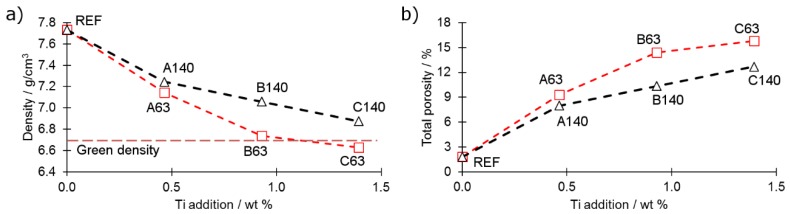
(**a**) Density changes and (**b**) porosity in function of the added titanium of Ø20 × 5 mm samples.

The results of dilatometric tests (Figure 3c) correspond well with the density of sintered samples (Figure 4) in respect to the direction of changes. There is, however, no agreement between these results in respect to the magnitude of changes. The shrinkage observed in dilatometric samples was heavily impaired by the evaporation of the boron from the sample surface [22]. Missing boron resulted in creation of smaller amount of eyectic phase. This effect is the more pronounced the smaller is the sample and the larger is its surface. As can be easily seen, the higher the addition of titanium, the lower the density after the sintering process. This effect was more pronounced for the 45–63 µm titanium particle size compared to the size of 100–140 µm. This shows that the reaction between the titanium and the eutectic phase was more effective when fine particles of high specific surface area were added.

### 3.3. Phase Identification

**Figure 5:**
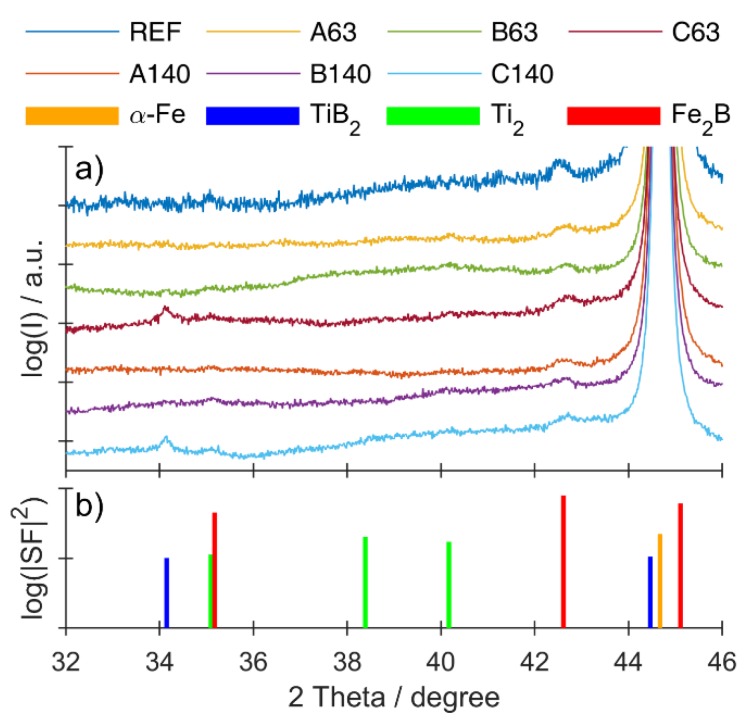
(**a**) Specular X-ray diffraction patterns of the Fe-B system with titanium additions; (**b**) expected peak positions from known crystal structures and their square of the absolute value of the structure factor SF as a measure for expected intensity.

Results of specular X-ray diffraction experiments are shown in Figure 5a together with peak positions from literature in Figure 5b. As a measure for expected intensity, the absolute value of the squared structure factor is used. For clarity, the experimental curves are shifted vertically. A major Bragg peak can be observed at 2 Theta = 44.7°, corresponding to the 110 reflex of the cubic iron phase (a = 2.8665 Å) [23]. Additional peaks of this phase can be found at 65.1°, 82.4°, 99.0° and 116.5°, originating from the (200), (211), (220) and (310) planes (Figure S1 in supplementary data). In the reference sample as well as all samples with titanium addition, additional peaks due to the presence of Fe_2_B can be observed (35.1° and 42.6°, corresponding to the 200 and 002 plane) [24]. At high titanium concentrations, a simultaneous presence of these peaks and the peak of the 100 reflex of TiB_2_ [25] can be found. Such observations stay in a good agreement with results of [17,18] who observed the formation of TiB_2_ at the expense of Fe_2_B borides in cast iron-based alloys during the solidification process. Moreover, a weak presence of crystalline titanium (40.2°) was noticed, informing that the reaction between titanium and boron was not complete [23].

**Figure 6:**
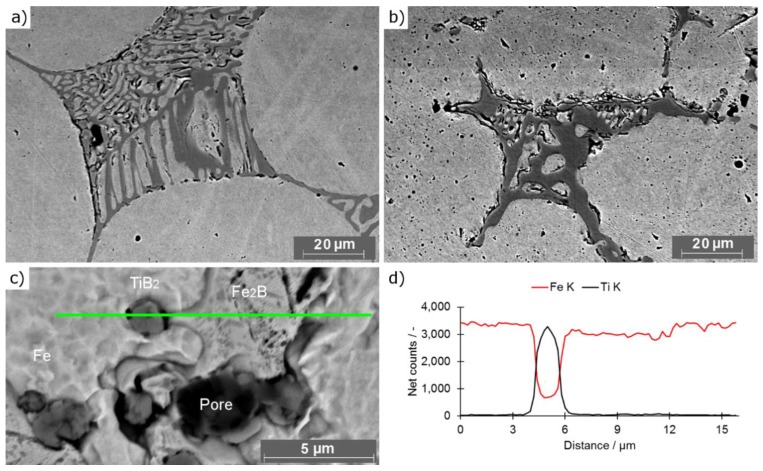
SEM pictures of borides: (**a**) REF sample; (**b**) C63 sample; (**c**) TiB_2_ neighbouring the Fe_2_B; (**d**) EDS line scan of sample C63 presented in Figure 6c.

Comparison of observed borides (REF and C63 samples) was presented in Figure 6a,b. Figure 6a shows borides in a network of rib-like structures observed in REF sample, whereas Figure 6b shows interconnected globular structures observed in titanium modified sintered compacts. This change in morphology was caused by crystallization of TiB_2_ as a result of the reaction of titanium and boron. The crystallisation process requires binding boron from a eutectic liquid resulting in precipitation of iron atoms in surrounding space. As the TiB_2_ is solid at sintering temperature it consisted a barrier for grain growth. This can be seen as uneven grain boundaries in samples with titanium addition (Figure 6b). Without the presence of titanium, Fe_2_B crystallizes at lower temperatures as the last phase (Figure 1a), therefore its morphology is determined by already existing spherical grains. Ex situ SEM EDX measurements (Figure 6c,d) after the sintering process showed the presence of small and nearly spherical titanium based particles in all samples with titanium additions. Moreover, the addition of titanium causes separation of previously (REF) connected borides, which enhances the connection among neighbouring grains (figures available in supplementary data in the folder: Eutectics distribution).

**Figure 7:**
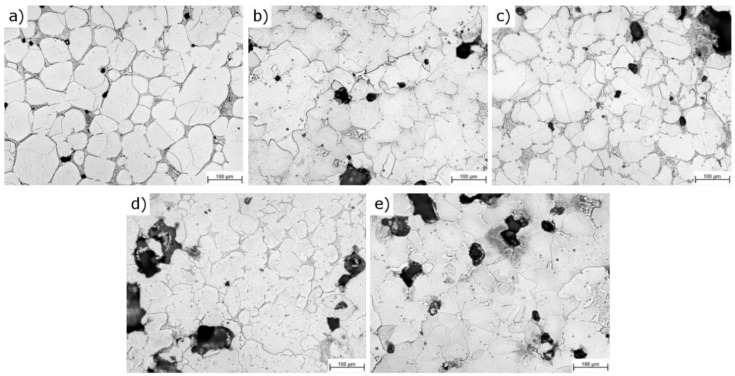
Representative microstructure micrographs of the tested samples: (**a**) REF; (**b**) A63; (**c**) A140; (**d**) C63; (**e**) C140 (detailed micrographs in supplementary data).

Furthermore, the titanium addition influences also the microstructure of the sintered material as presented in Figure 7a–e. The reference sample characterizes with a nearly continuous network of borides located on grain boundaries and uniaxial grains. Contrary to the REF sample, the more titanium was introduced, the more developed the grains’ shapes became. The presence of the TiB_2_ in the matrix of the material may effectively hinder the grain growth, as was demonstrated in research carried out, e.g., by Namini et al., Sobhani et al. and Gan et al. [26,27,28]. Moreover, the more titanium was added, the more and the larger pores were observed. This is believed was a side effect of limiting the amount of liquid phase during the sintering process.

**Figure 8:**
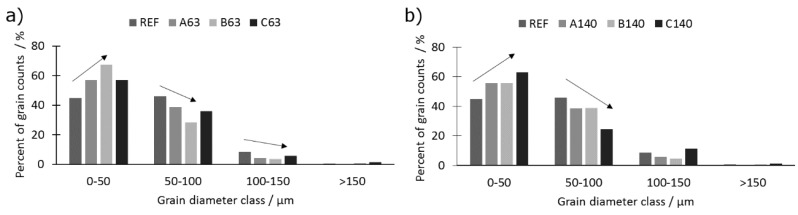
Histograms of grain size distribution for series (**a**) 63 and (**b**) 140.

A comparison of a grain size presented in Figure 8 shows that the introduction of titanium of particle size of 63 μm (Figure 8a) results in grain refinement, which stays in good agreement with other researchers’ findings [26,27,28]. A similar trend was observed when titanium was introduced in the form of bigger particles (140 μm). The grain size refinement is caused by the presence of a TiB_2_, which consists of an effective barrier for grain growth.

The presence of TiB_2_ (induced by addition of Ti) changed also the grain shape, i.e., grains in REF sample were of polygonal shape with rounded corners due to presence of large amount of borides. Moreover, the more titanium was added, the more irregular was the shape of the grains and the more dispersed were the borides. Furthermore, as appears from the analysis of grain size distribution a large amount of titanium (1.40 wt % – C) promotes also the appearance of large grains (diameter 100–150 μm) in the case of series 140. The medium-sized (50–100 μm) grains are promoted when titanium is added in the form of a particle of size 63 μm.

### 3.4. Mechanical Properties

**Figure 9:**
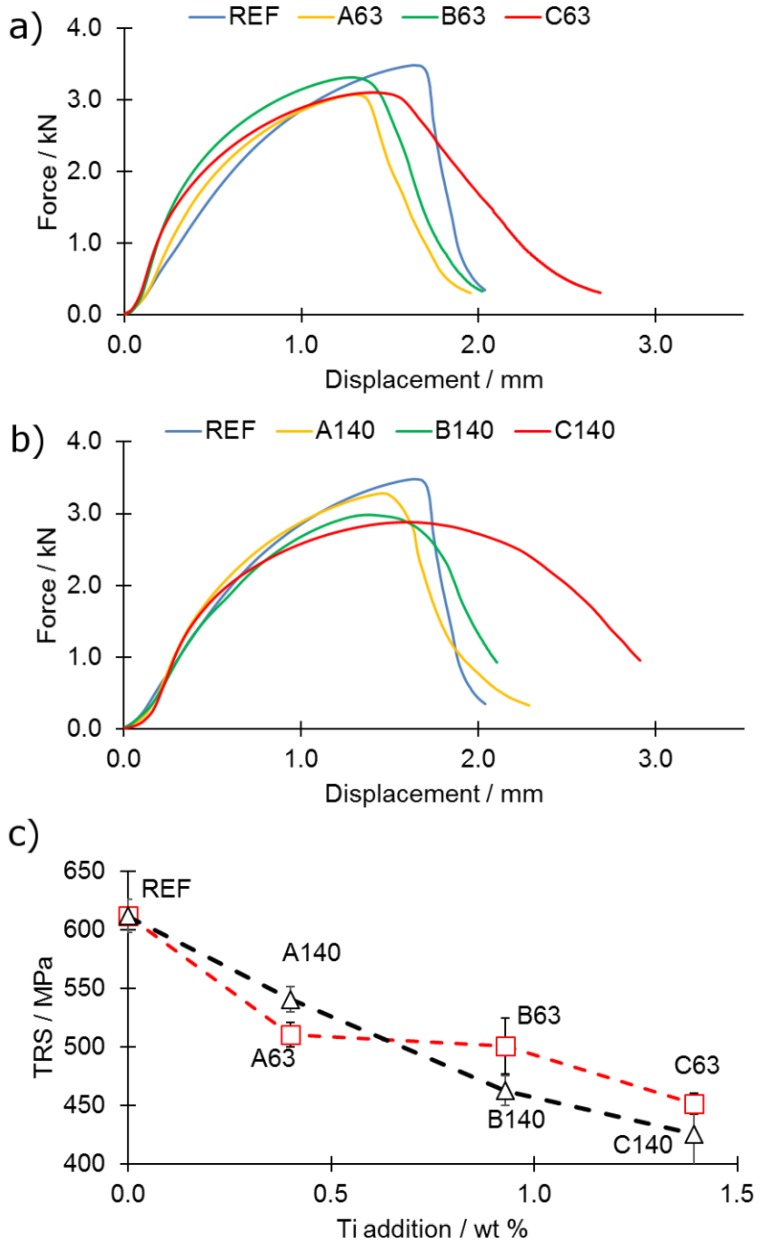
Representative Transverse Rupture Strength (TRS) curve for samples from series (**a**) 63 and (**b**) 140; (**c**) comparison of TRS values of tested samples.

Transverse Rupture Strength (TRS) tests were performed in order to estimate the influence of the changed phase composition on the mechanical behaviour of the samples. The results were presented in a way to highlight the influence of the increasing amount of titanium addition on mechanical behaviour (Figure 9a–c). The results bring the conclusion that high addition of titanium (C-series, 1.40 wt % Ti) causes an increase in the maximum observed deformation on the expense of maximum force (Figure 9a,b) and hence, the TRS as well (Figure 9c). Other additions of titanium (A and B) show intermediate states—samples with 0.47 wt % titanium addition (A) show a decrease of both TRS and maximum deformation, whereas the B-series (0.93 wt % titanium addition) is characterized by lower peak force and higher maximum deformation with respect to the reference sample (Figure 9). The increase of ductility remains in the agreement of the other works [17,18]. The drop of the mechanical properties, however, stays in opposition to the recent findings [17,18]. The increase in ductility of sample (C series) can be attributed to joint effect of few phenomena: (i) the grains are not anymore separated with a network of brittle borides; therefore, grains are connected with each other; (ii) formation of TiB_2_ reinforces the matrix; (iii) grains are not uniaxial anymore and their shape is irregular; therefore, mutual interlocking is possible. The irregular shape of grains is attributed to the joint effect of grain growth in conditions of solid-state sintering (induced by eutectic phase disappearance) and liquid phase sintering. These changes were possible mainly due to the change of manufacturing process change—in previous works, the casting was used whereas is the present work the liquid phase sintering process was applied. For the densification of the sample, effective mass transport is required, and when the titanium is added, it is impeded as presented in Figure 4, which leads to the drop of the density (in relation to the REF sample).

**Figure 10:**
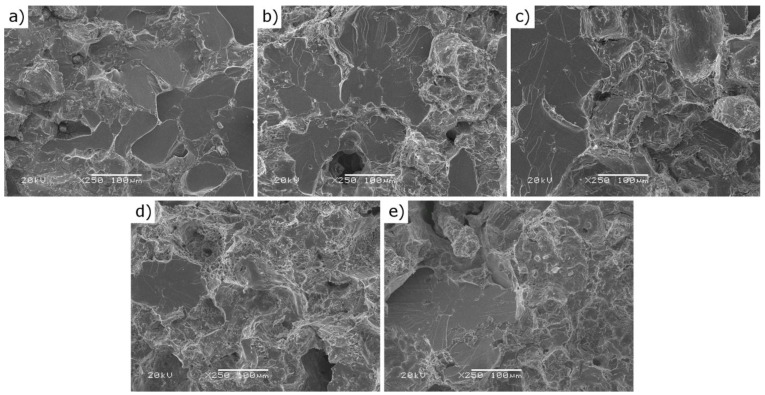
Representative fracture surfaces of the tested samples: (**a**) REF; (**b**) A63; (**c**) A140; (**d**) C63; (**e**) C140.

Fracture surfaces investigated using SEM (Figure 10) reveal that the more titanium was added the more ductile was the behaviour of the fracture surfaces, which can be seen by the development of the fracture surface in Figure 10a–e. The more titanium was introduced the less flat intra-granular fractures were observed and the more signs of ductility were present, i.e., “mountain-chain shapes and valleys”. The extent of the changes was relatively small. This, however, suggests a lesser amount of precipitated brittle borides at the grains boundaries compared to the material without titanium addition (REF).

## 4. Conclusions

The influence of increasing titanium addition on the iron-boron system was under investigation. Titanium was introduced in three various amounts of two different grain size distributions each. It was shown that the addition of titanium partially binds the boron from the Fe_2_B + Fe eutectic liquid phase and, therefore, reduces the shrinking rate by influencing the amount of the eutectic liquid phase. It was also shown that the addition of titanium changes the phase composition of the final material by creating crystalline TiB_2_ borides besides crystalline Fe_2_B borides. Changes in the morphology from a network of rib-like structures for the pure Fe-B system to interconnected globular structures in case of titanium additions were also observed due to TiB_2_ appearance. Both, increasing amount of titanium addition and its fine grain size distribution, impede the shrinking of Fe-B system by decreasing the amount of liquid phase. The addition of titanium enhances the plastic behaviour of a sintered material at the expense of transverse rupture strength. This change was attributed to (i) grain refinement; (b) irregular shape of grains, which allowed for mutual interlocking, and (iii) decreasing amount of brittle borides on the grain boundaries, which enhanced the connection among the neighbouring grains.

Contrary to the casting process, the sintering process was found to be not suitable for strengthening iron-based sintered compacts via TiB_2_ in situ formation due to a negative influence on the densification behaviour of the sintered material.

## Figures and Tables

**Table 1:** Composition of utilised powder blends.

Sample Description	Titanium Grain Size Class/μm	Titanium/wt %	Boron/wt %	Iron/wt %
REF	0	0.00	0.80	balance
A63	45–63	0.47		
B63	45–63	0.93		
C63	45–63	1.40		
A140	100–140	0.47		
B140	100–140	0.93		
C140	100–140	1.40		

## Supplementary Materials

The following are available online at https://www.mdpi.com/1996-1944/12/24/4188/s1, Figure S1: Specular X-ray diffraction patterns of the Fe-B system with titanium additions over the full measured range. Grey lines denote expected peak positions of the cubic iron phase.
