# Supplementary material for: In Situ Formation of TiB2 in Fe-B System with Titanium Addition and Its Influence on Phase Composition, Sintering Process and Mechanical Properties
Source: Materials (Basel). 2019 Dec 13;12(24):4188. doi: 10.3390/ma12244188 (PMC6947460; doi:10.3390/ma12244188)
Supplement: Supplementary file 1 [file materials-12-04188-s001.zip › materials-566283-supplementary/Final supplementary/Figure S1.docx]

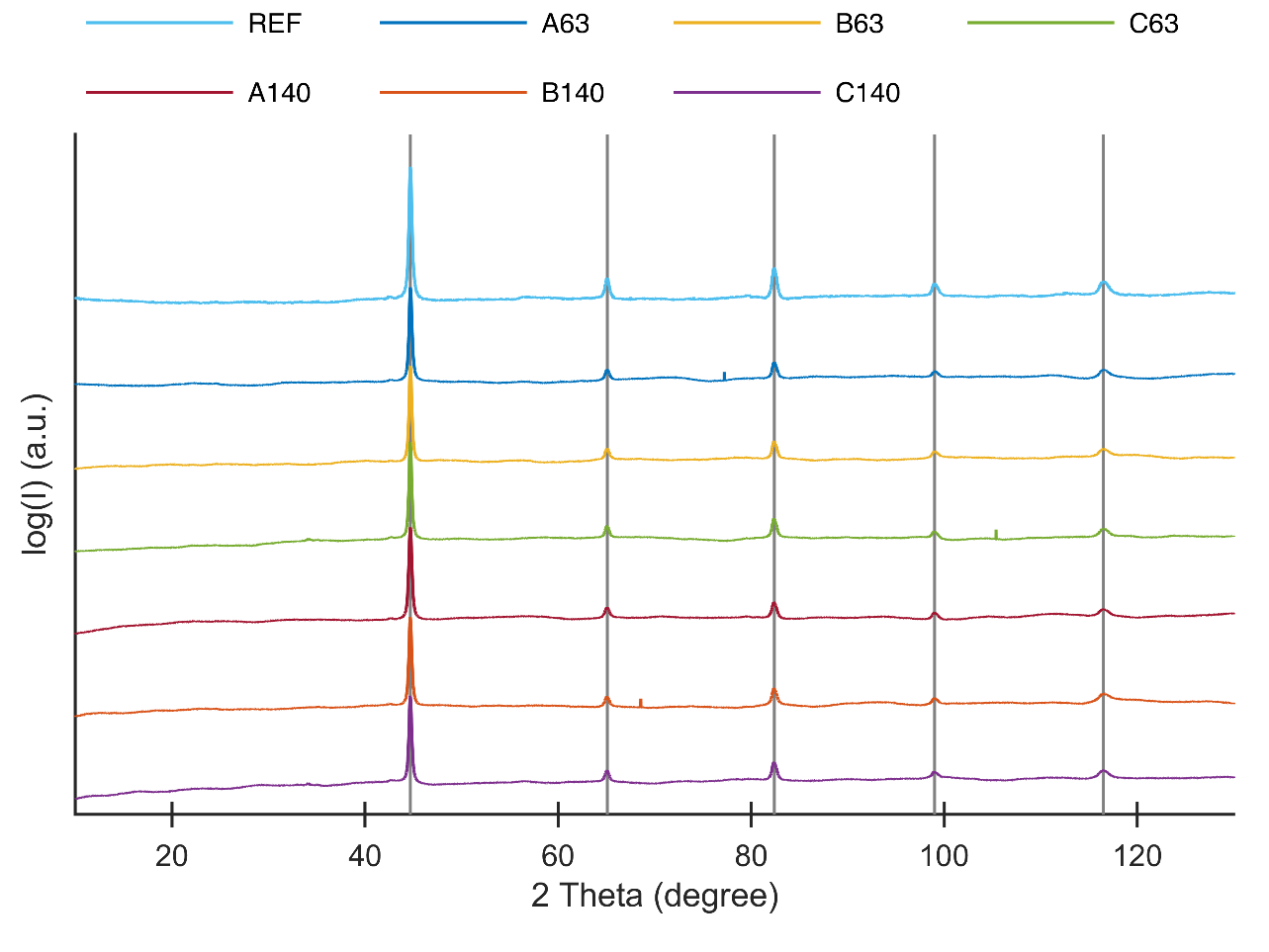


**Figure S1.** Specular X-ray diffraction patterns of the Fe-B system with titanium additions over the full measured range. Grey lines denote expected peak positions of the cubic iron phase.
