# Supplementary material for: In Situ Formation of TiB2 in Fe-B System with Titanium Addition and Its Influence on Phase Composition, Sintering Process and Mechanical Properties
Source: Materials (Basel). 2019 Dec 13;12(24):4188. doi: 10.3390/ma12244188 (PMC6947460; doi:10.3390/ma12244188)
Supplement: Supplementary file 1 [file materials-12-04188-s001.zip › materials-566283-supplementary/Final supplementary/Iron certificate.pdf]

SIGMA-ALDRICH®

3050 Spruce Street, Saint Louis, MO 63103 USA
Email USA: techserv@sial.com Outside USA: eurtechserv@sial.com

Certificate of Analysis

**Product Name:**	IRON<br>>= 99 %, reduced, powder fine
**Product Number:**	12310
**Batch Number:**	STBJ0776
**Brand:**	Aldrich
**CAS Number:**	7439-89-6
**Formula:**	Fe
**Formula Weight:**	55.85
**Quality Release Date:**	04 MAR 2019
**Recommended Retest Date:**	AUG 2023

TEST	SPECIFICATION	RESULT
ASSAY	≥ 99 %	99.6 %
INSOLUBLE MATTER	≤ 0.5 % (IN HCL)	< 0.1 %
ARSEN	≤ 0.0005 %	< 0.0005 %
COPPER	≤ 0.01 %	< 0.01 %
MANGANESE	≤ 0.1 %	< 0.1 %
NICKEL	≤ 0.05 %	< 0.05 %
LEAD	≤ 0.002 %	< 0.002 %
ZINC	≤ 0.005 %	< 0.005 %
CHLORIDE (CL)	≤ 0.002 %	< 0.002%
SULFIDE (S)	≤ 0.01 %	< 0.01 %

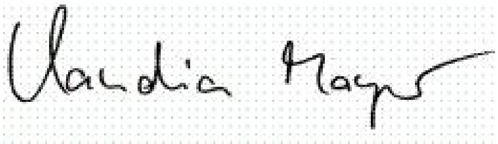

Claudia Mayer
Manager Quality Control
Steinheim, Germany

Sigma-Aldrich warrants that at the time of the quality release or subsequent retest date this product conformed to the information contained in this publication. The current specification sheet may be available at Sigma-Aldrich.com. For further inquiries, please contact Technical Service. Purchaser must determine the suitability of the product for its particular use. See reverse side of invoice or packing slip for additional terms and conditions of sale.
