# Supplementary figures and images for: In Situ Formation of TiB2 in Fe-B System with Titanium Addition and Its Influence on Phase Composition, Sintering Process and Mechanical Properties
Source: Materials (Basel). 2019 Dec 13;12(24):4188. doi: 10.3390/ma12244188 (PMC6947460; doi:10.3390/ma12244188)

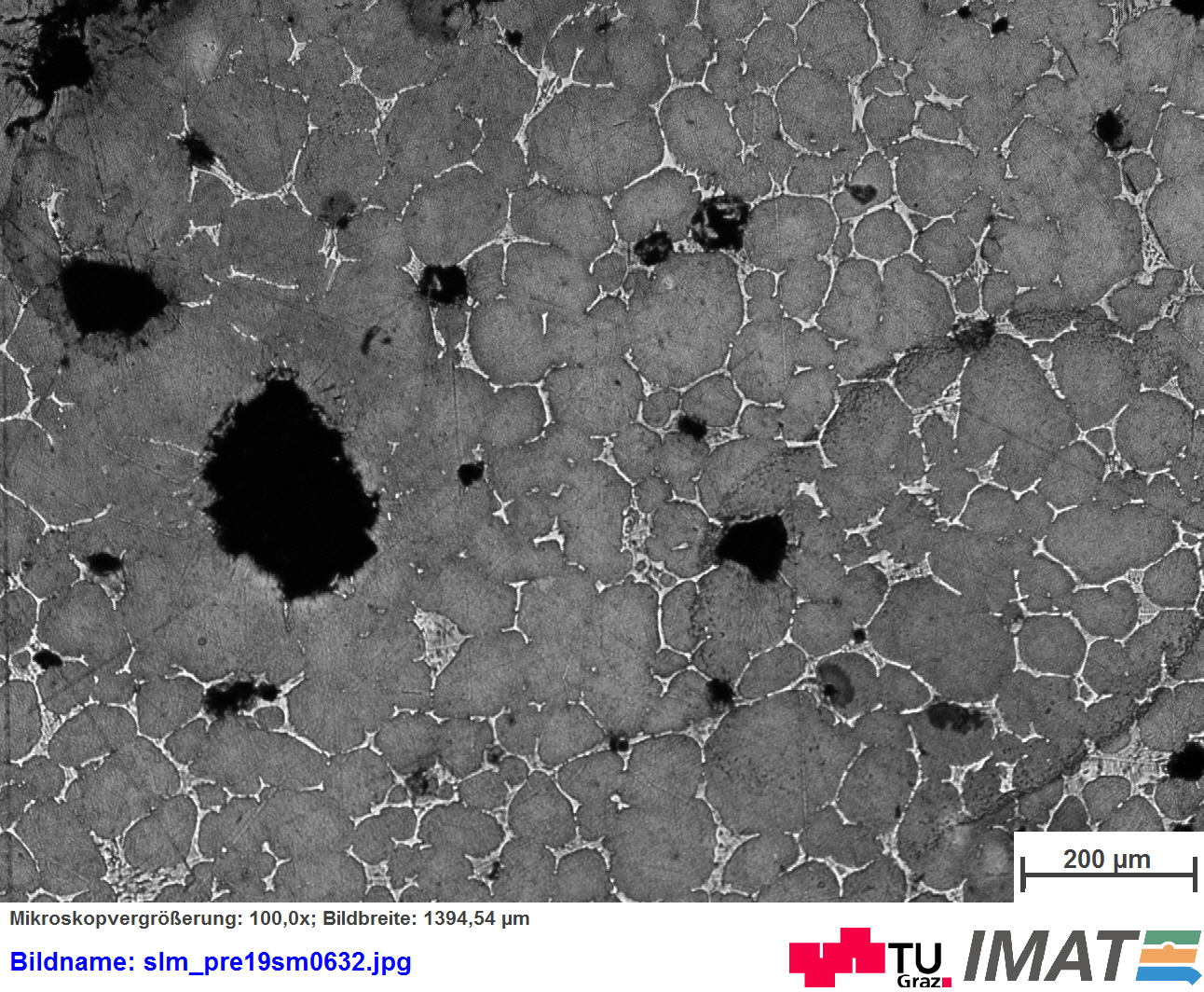

Supplement: Supplementary file 1 [file materials-12-04188-s001.zip › materials-566283-supplementary/Final supplementary/Eutectics distribution/A140 slm_pre19sm0632.jpg]

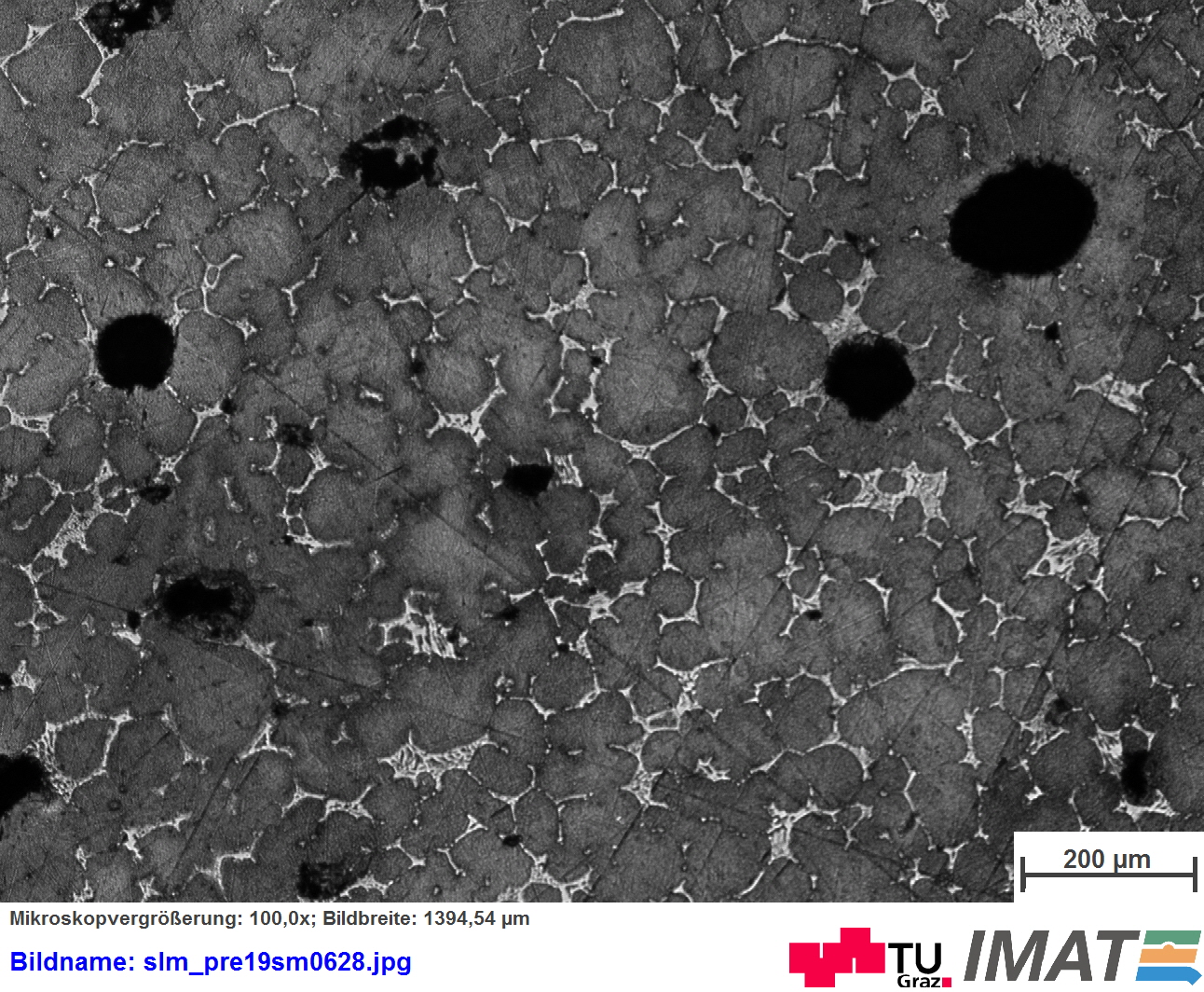

Supplement: Supplementary file 1 [file materials-12-04188-s001.zip › materials-566283-supplementary/Final supplementary/Eutectics distribution/A63 slm_pre19sm0628.jpg]

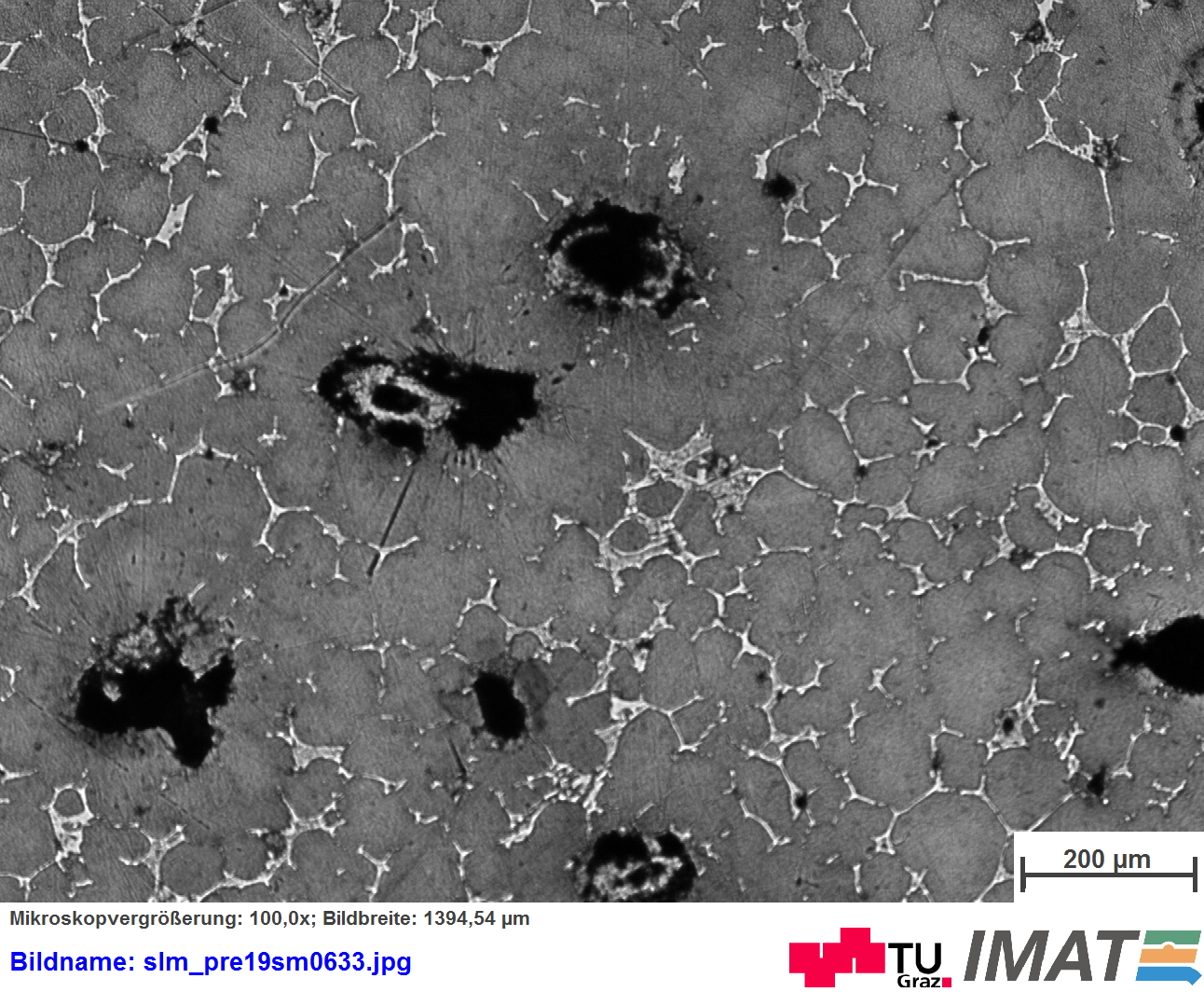

Supplement: Supplementary file 1 [file materials-12-04188-s001.zip › materials-566283-supplementary/Final supplementary/Eutectics distribution/B140 slm_pre19sm0633.jpg]

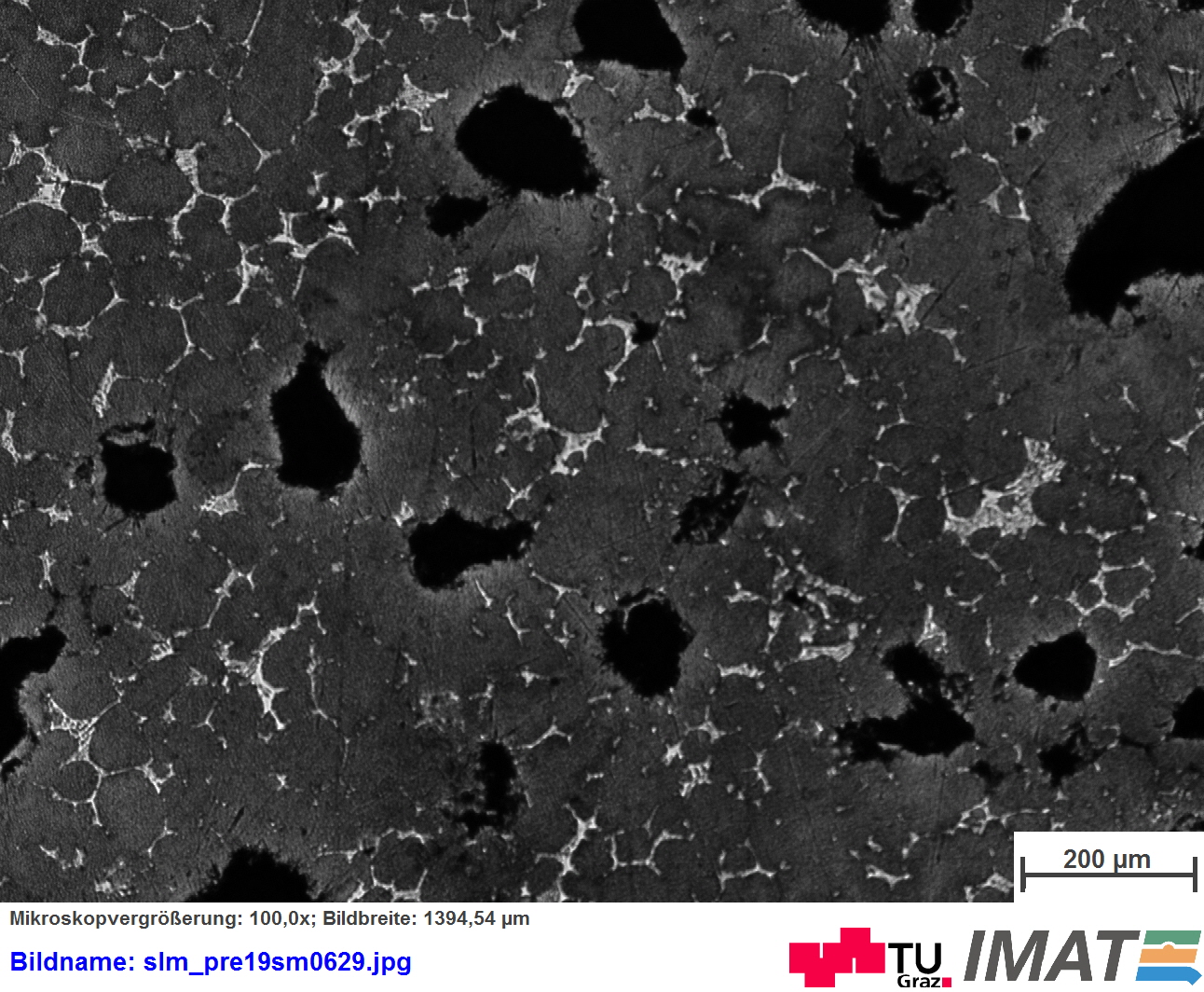

Supplement: Supplementary file 1 [file materials-12-04188-s001.zip › materials-566283-supplementary/Final supplementary/Eutectics distribution/B63 slm_pre19sm0629.jpg]

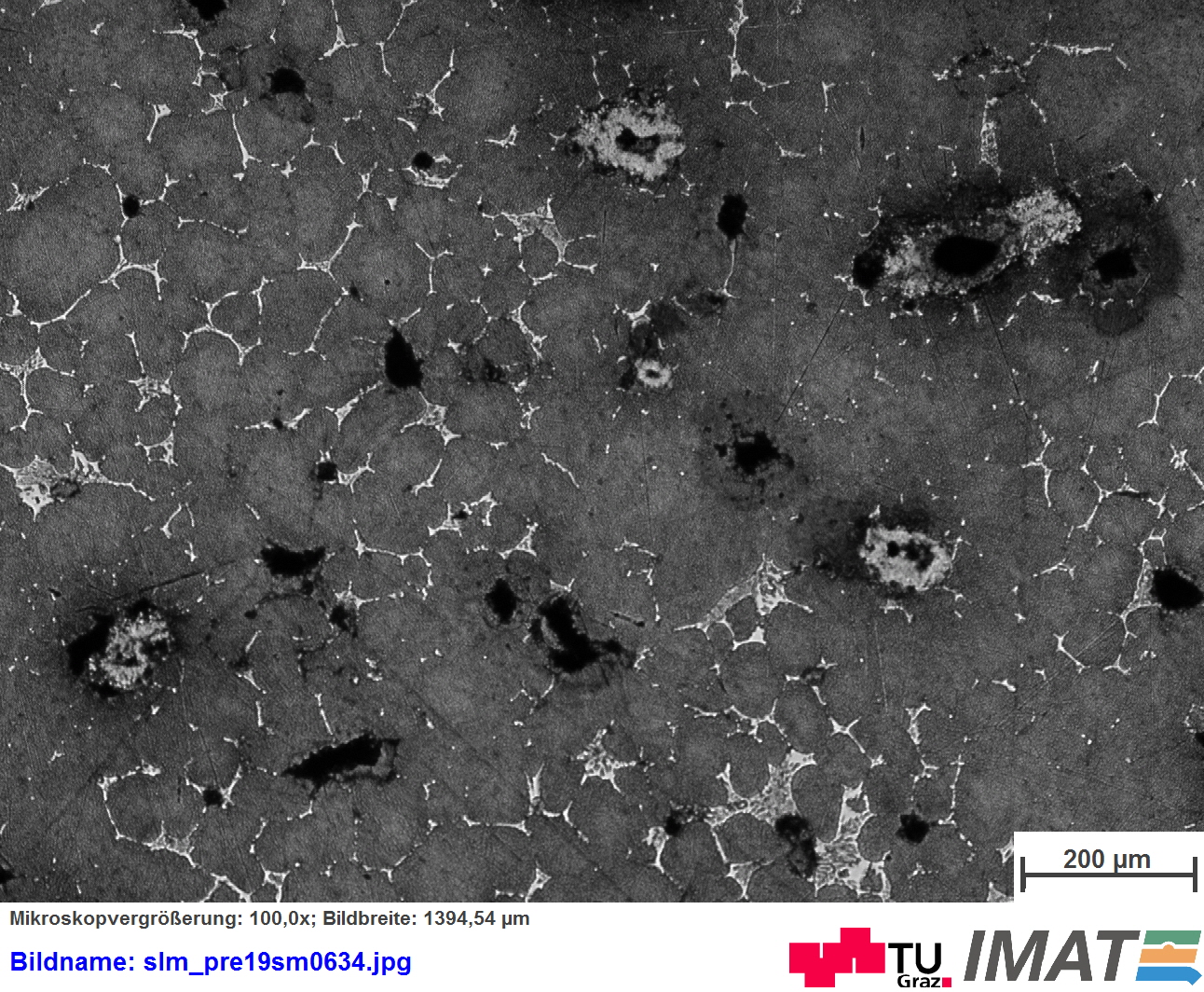

Supplement: Supplementary file 1 [file materials-12-04188-s001.zip › materials-566283-supplementary/Final supplementary/Eutectics distribution/C140 slm_pre19sm0634.jpg]

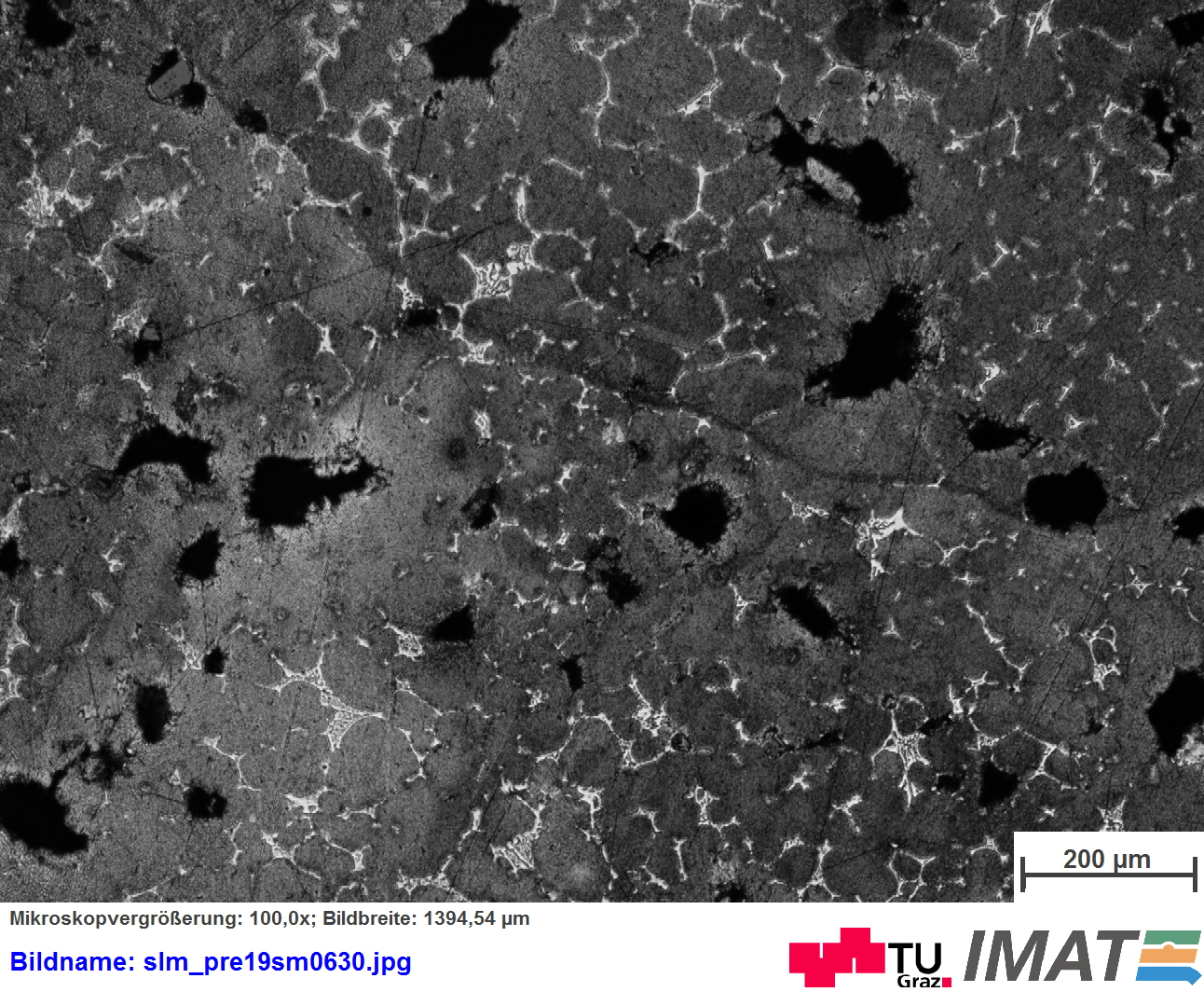

Supplement: Supplementary file 1 [file materials-12-04188-s001.zip › materials-566283-supplementary/Final supplementary/Eutectics distribution/C63 slm_pre19sm0630.jpg]

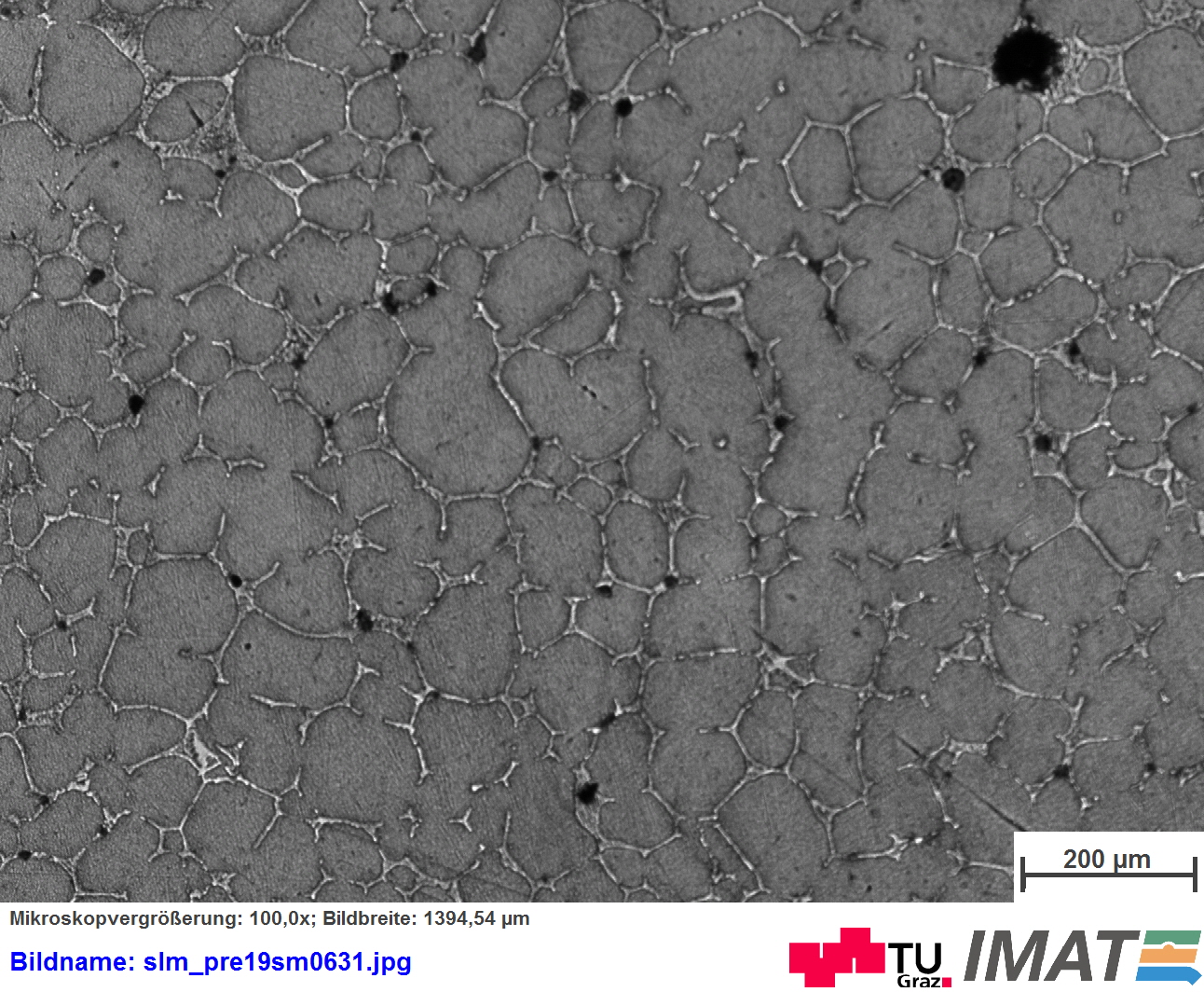

Supplement: Supplementary file 1 [file materials-12-04188-s001.zip › materials-566283-supplementary/Final supplementary/Eutectics distribution/REF slm_pre19sm0631.jpg]

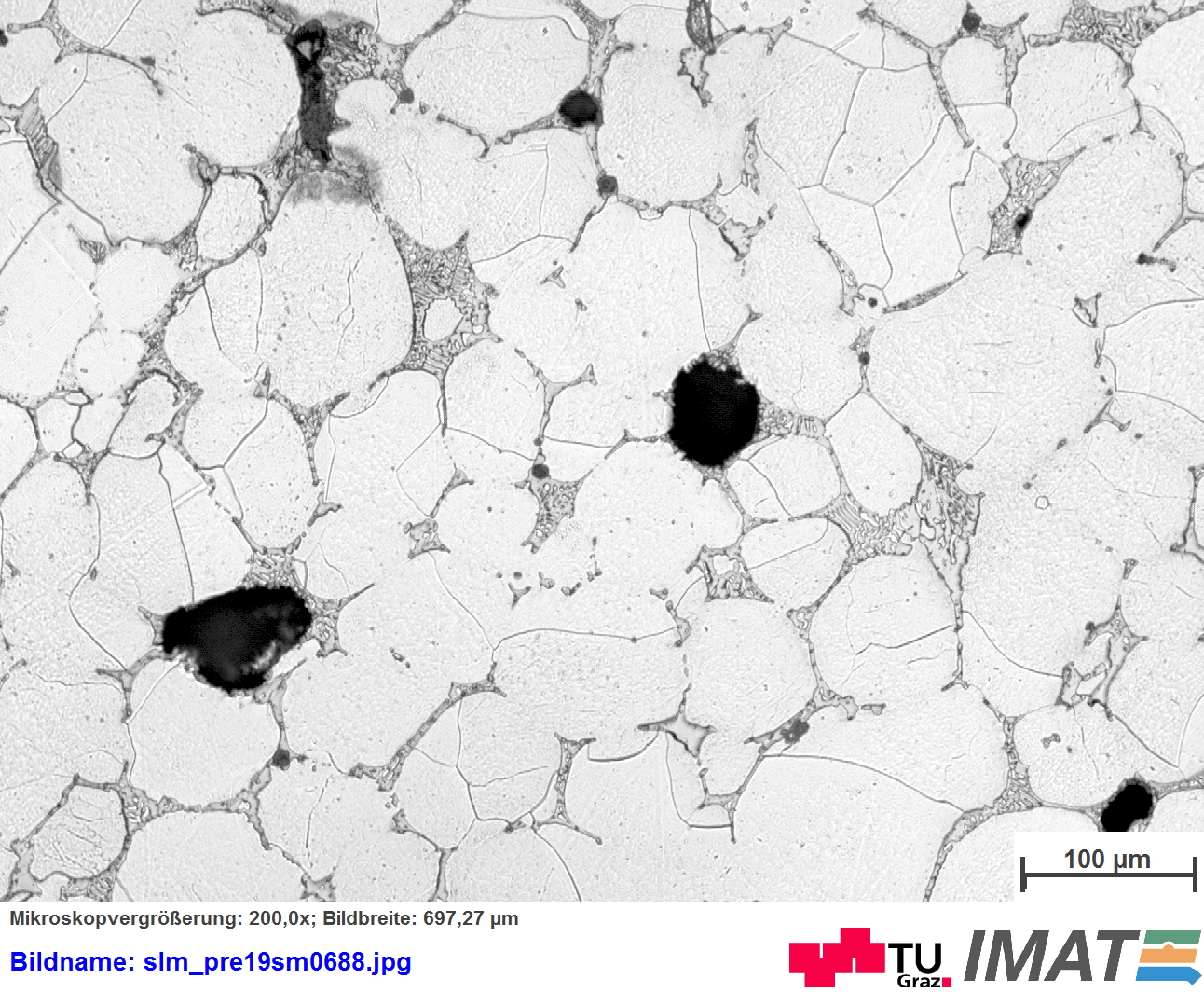

Supplement: Supplementary file 1 [file materials-12-04188-s001.zip › materials-566283-supplementary/Final supplementary/Microstrucutres/A140/slm_pre19sm0688.jpg]

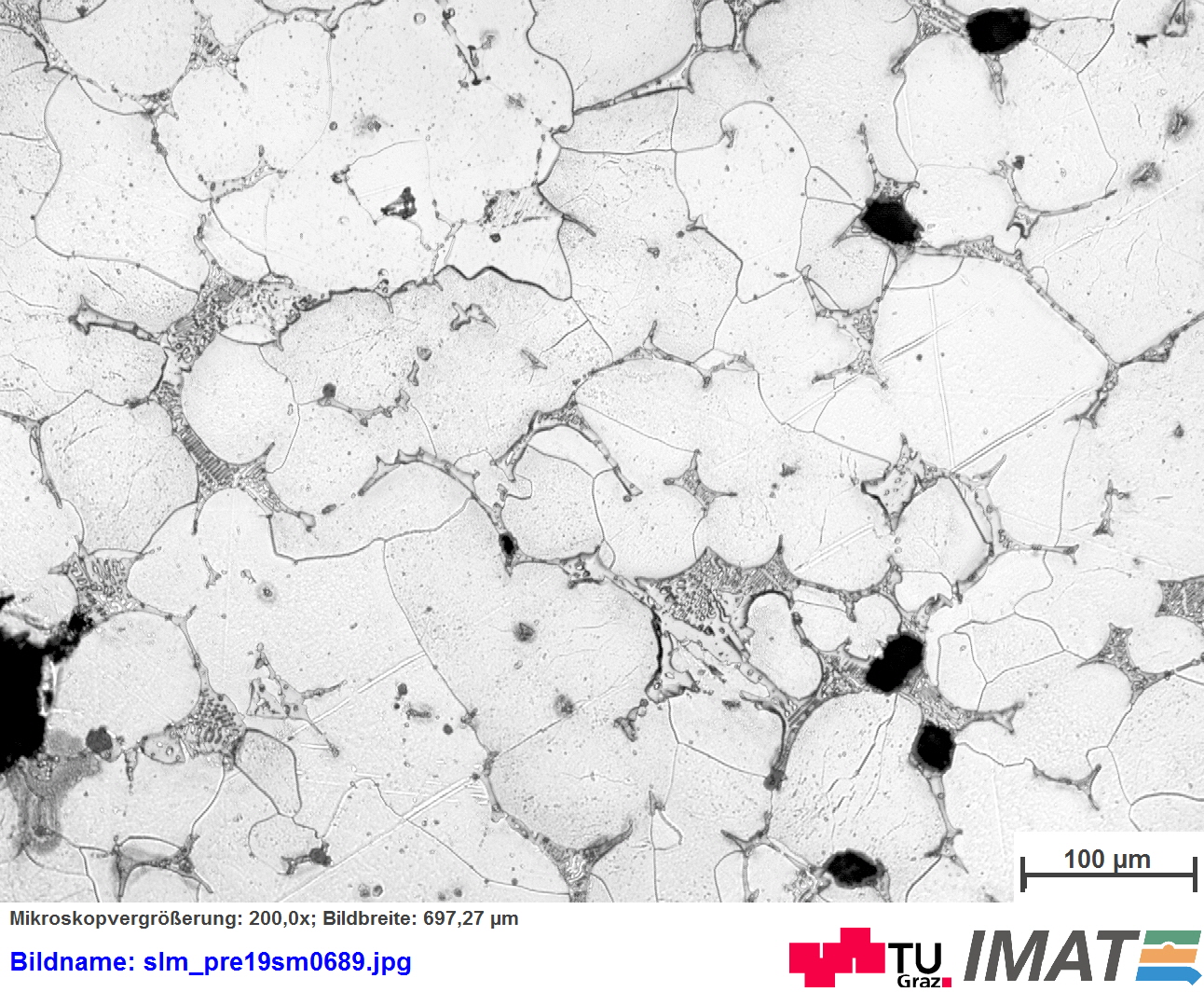

Supplement: Supplementary file 1 [file materials-12-04188-s001.zip › materials-566283-supplementary/Final supplementary/Microstrucutres/A140/slm_pre19sm0689.jpg]

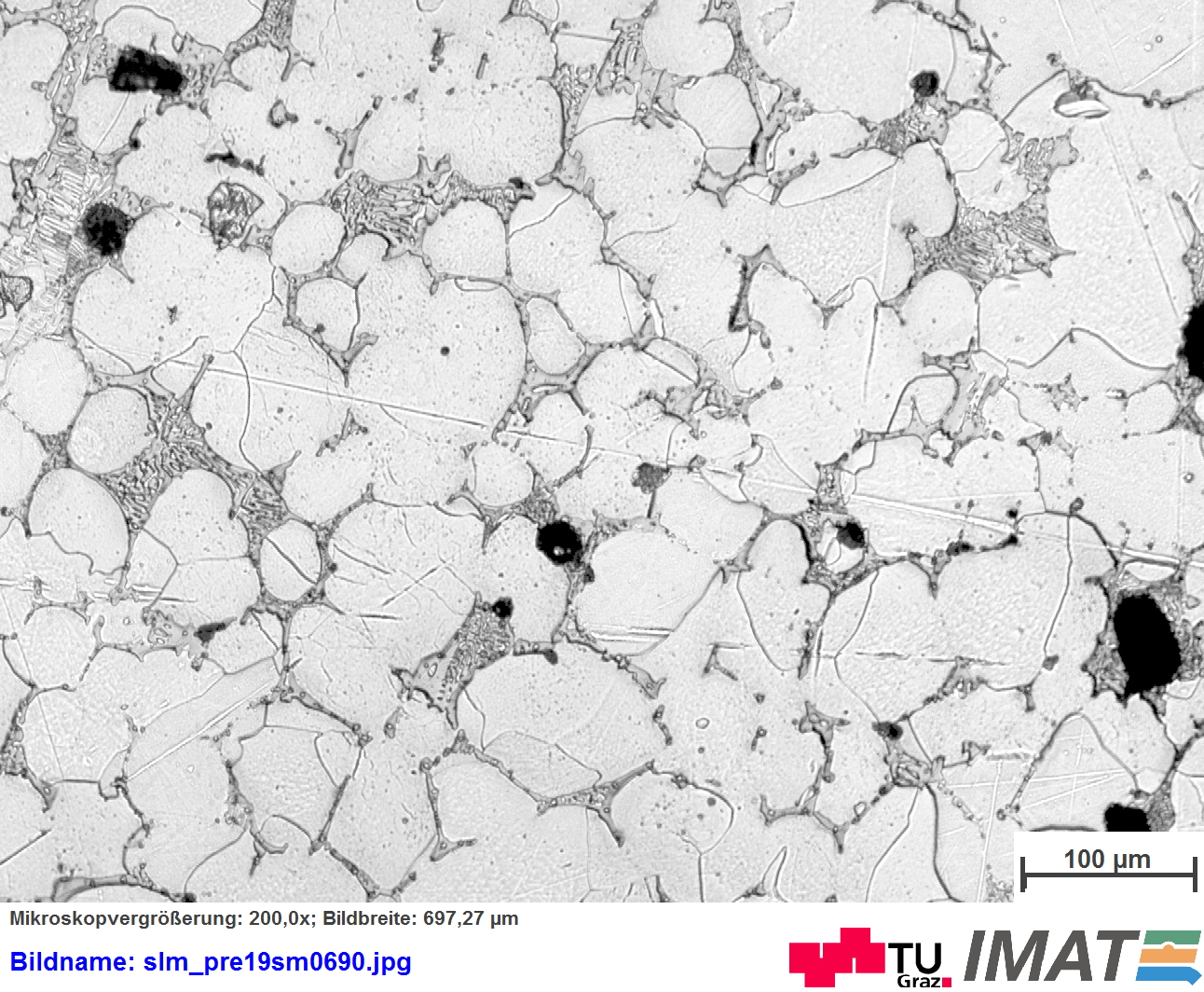

Supplement: Supplementary file 1 [file materials-12-04188-s001.zip › materials-566283-supplementary/Final supplementary/Microstrucutres/A140/slm_pre19sm0690.jpg]

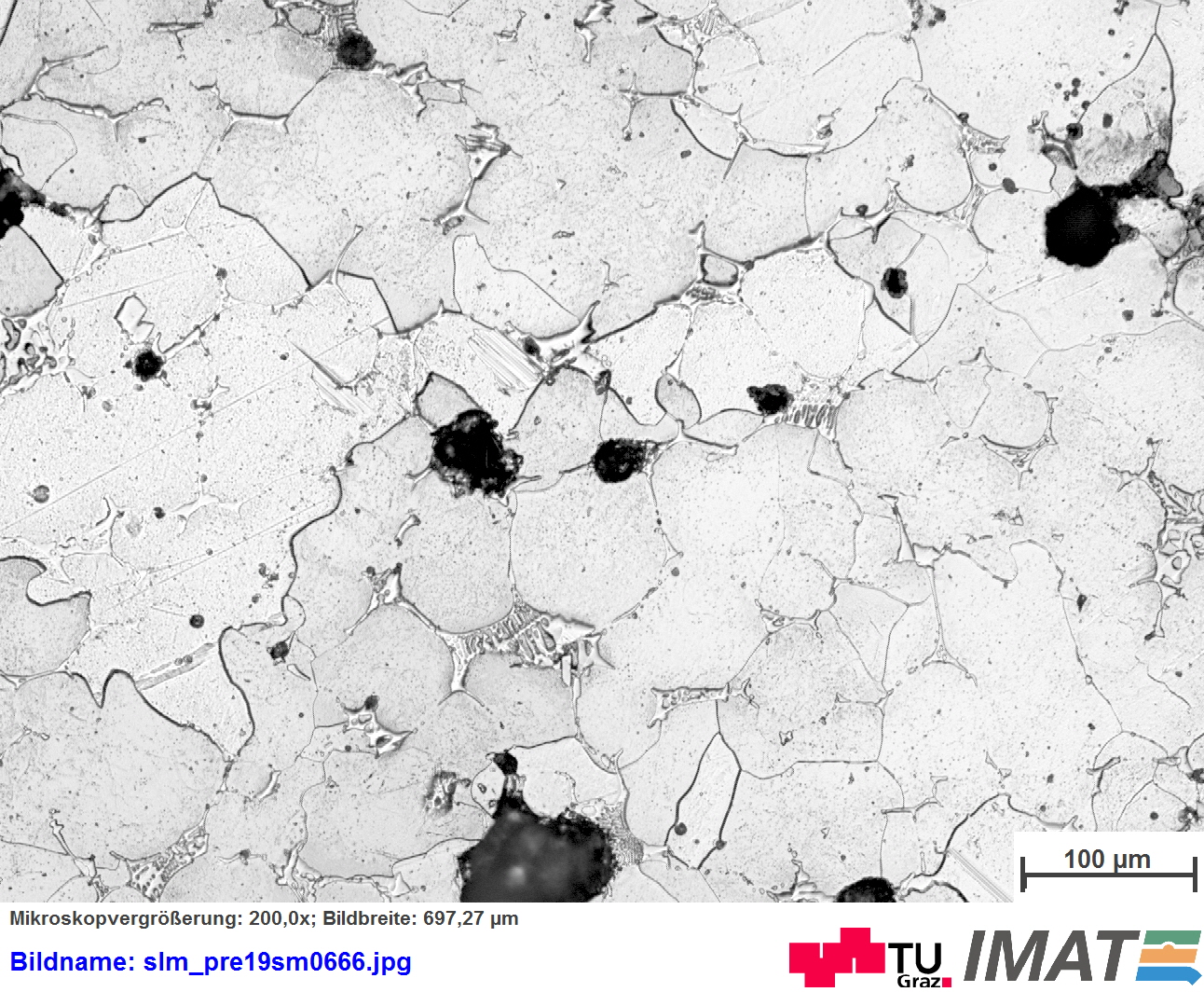

Supplement: Supplementary file 1 [file materials-12-04188-s001.zip › materials-566283-supplementary/Final supplementary/Microstrucutres/A63/slm_pre19sm0666.jpg]

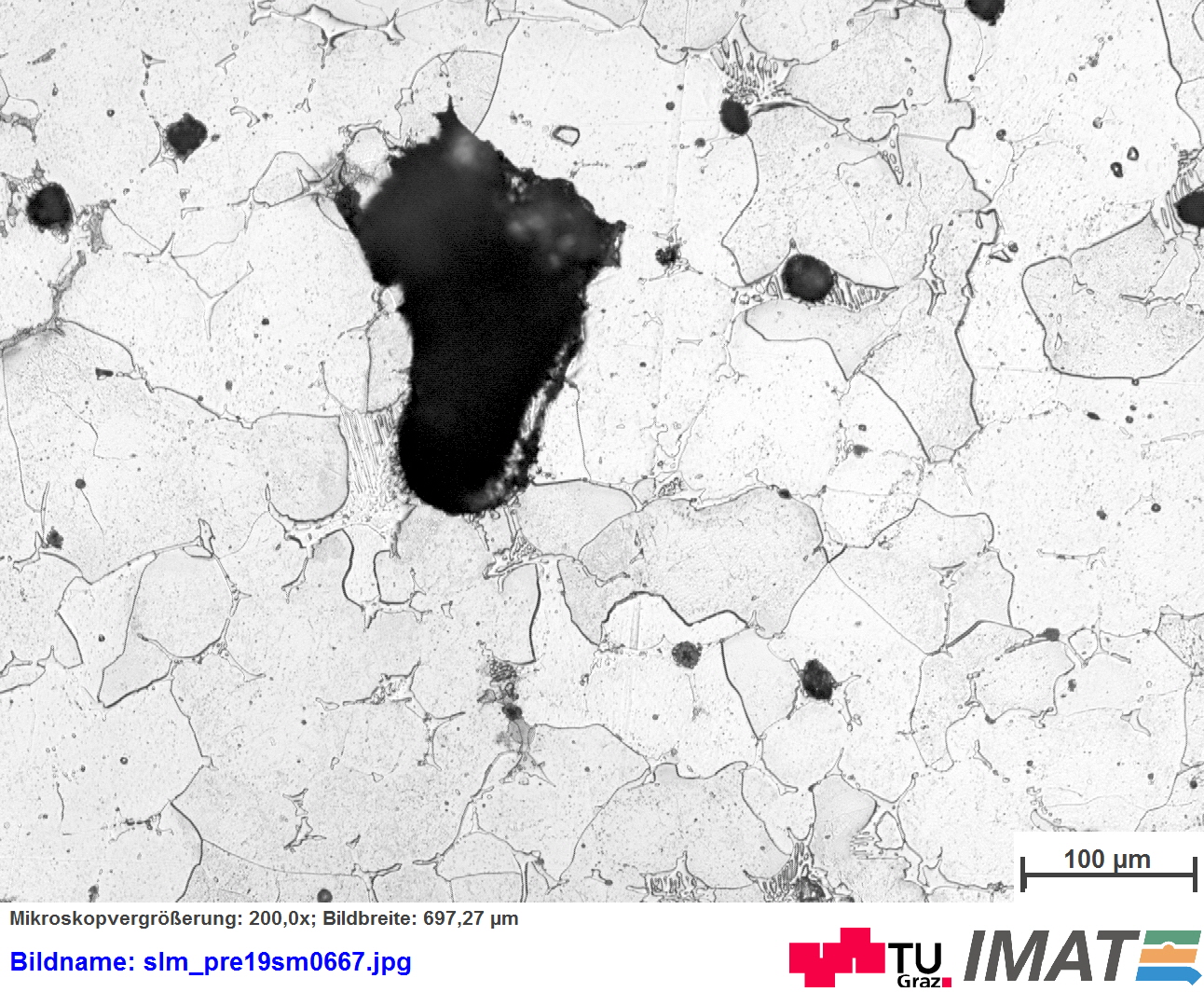

Supplement: Supplementary file 1 [file materials-12-04188-s001.zip › materials-566283-supplementary/Final supplementary/Microstrucutres/A63/slm_pre19sm0667.jpg]

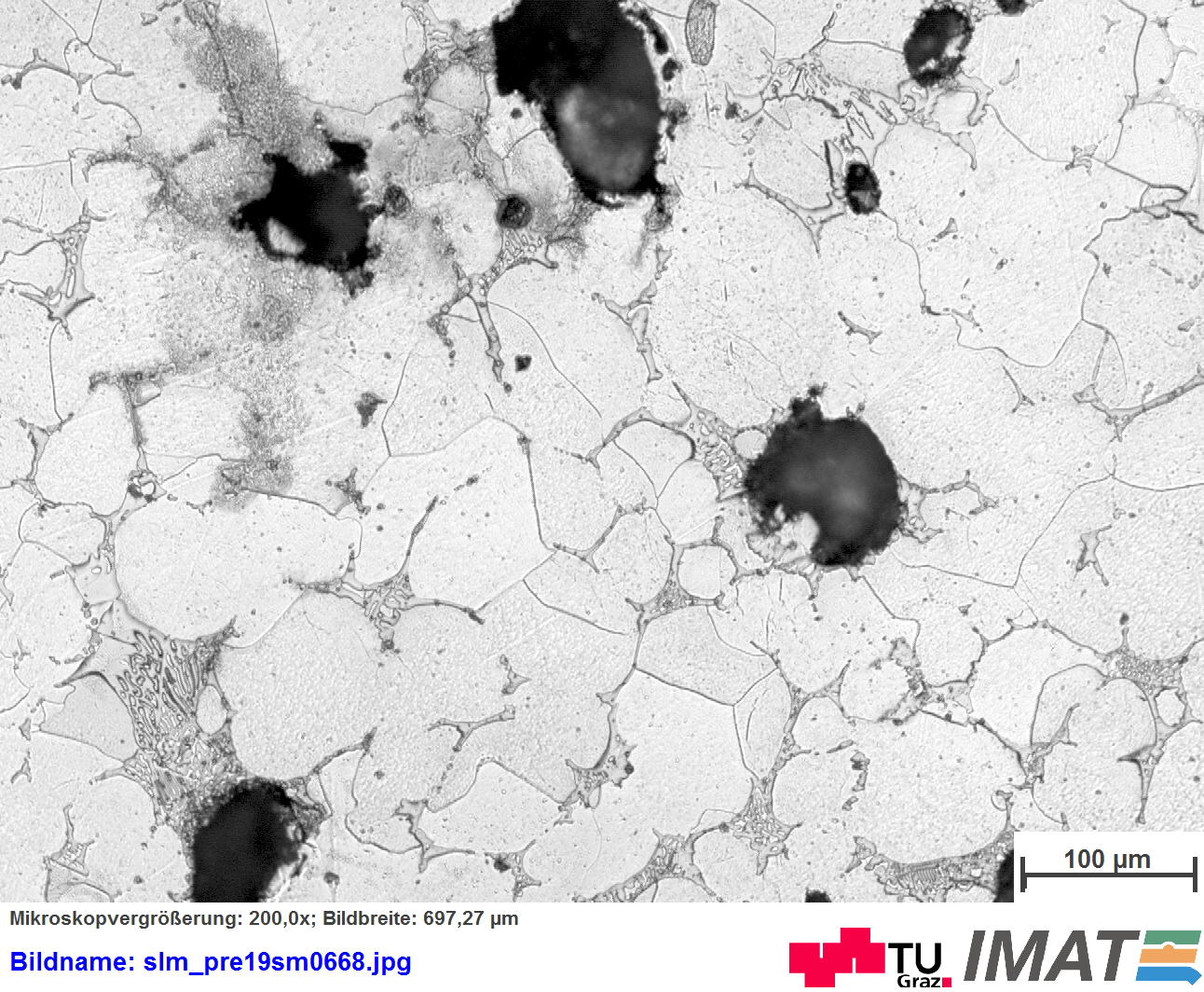

Supplement: Supplementary file 1 [file materials-12-04188-s001.zip › materials-566283-supplementary/Final supplementary/Microstrucutres/A63/slm_pre19sm0668.jpg]

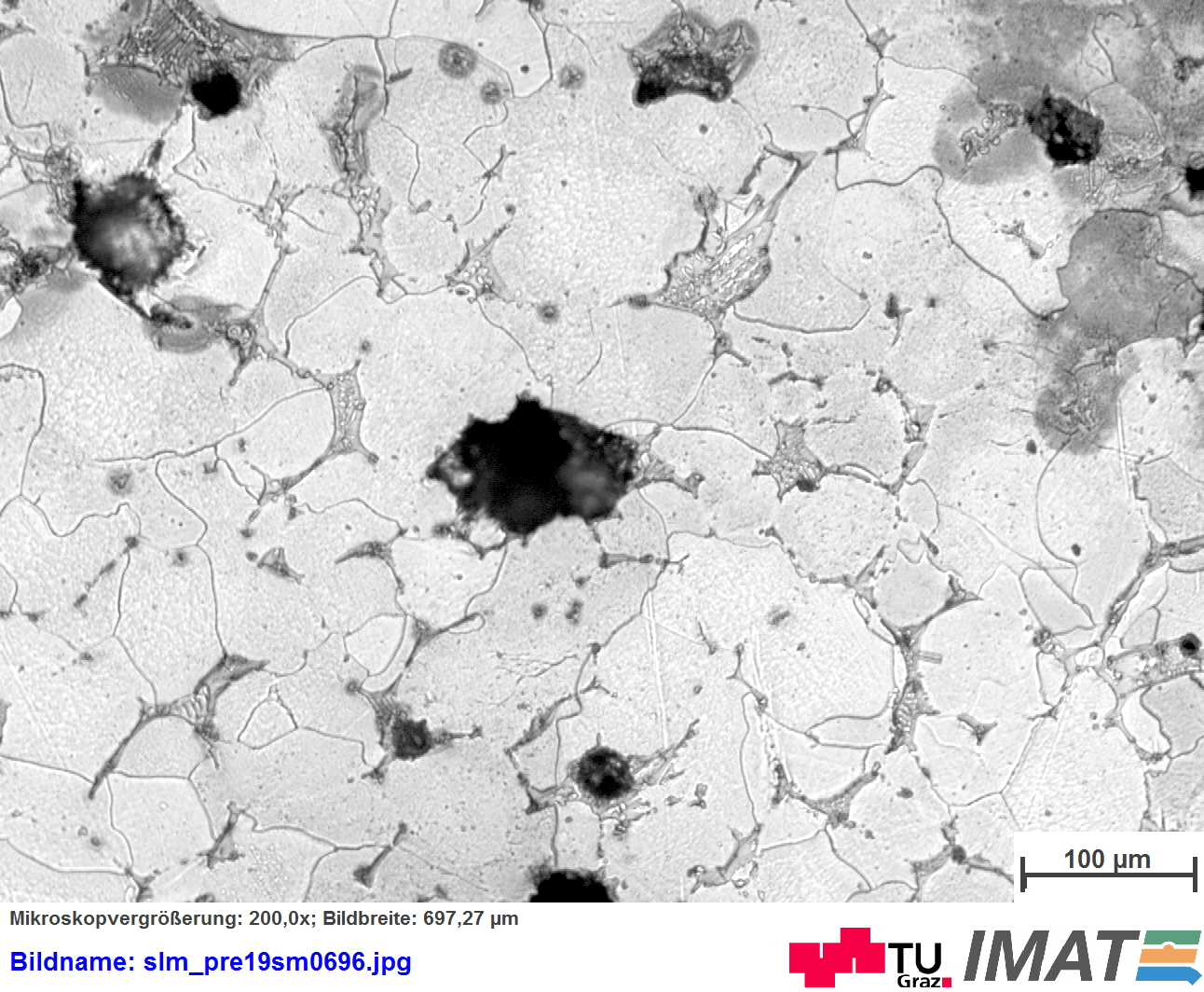

Supplement: Supplementary file 1 [file materials-12-04188-s001.zip › materials-566283-supplementary/Final supplementary/Microstrucutres/B140/slm_pre19sm0696.jpg]

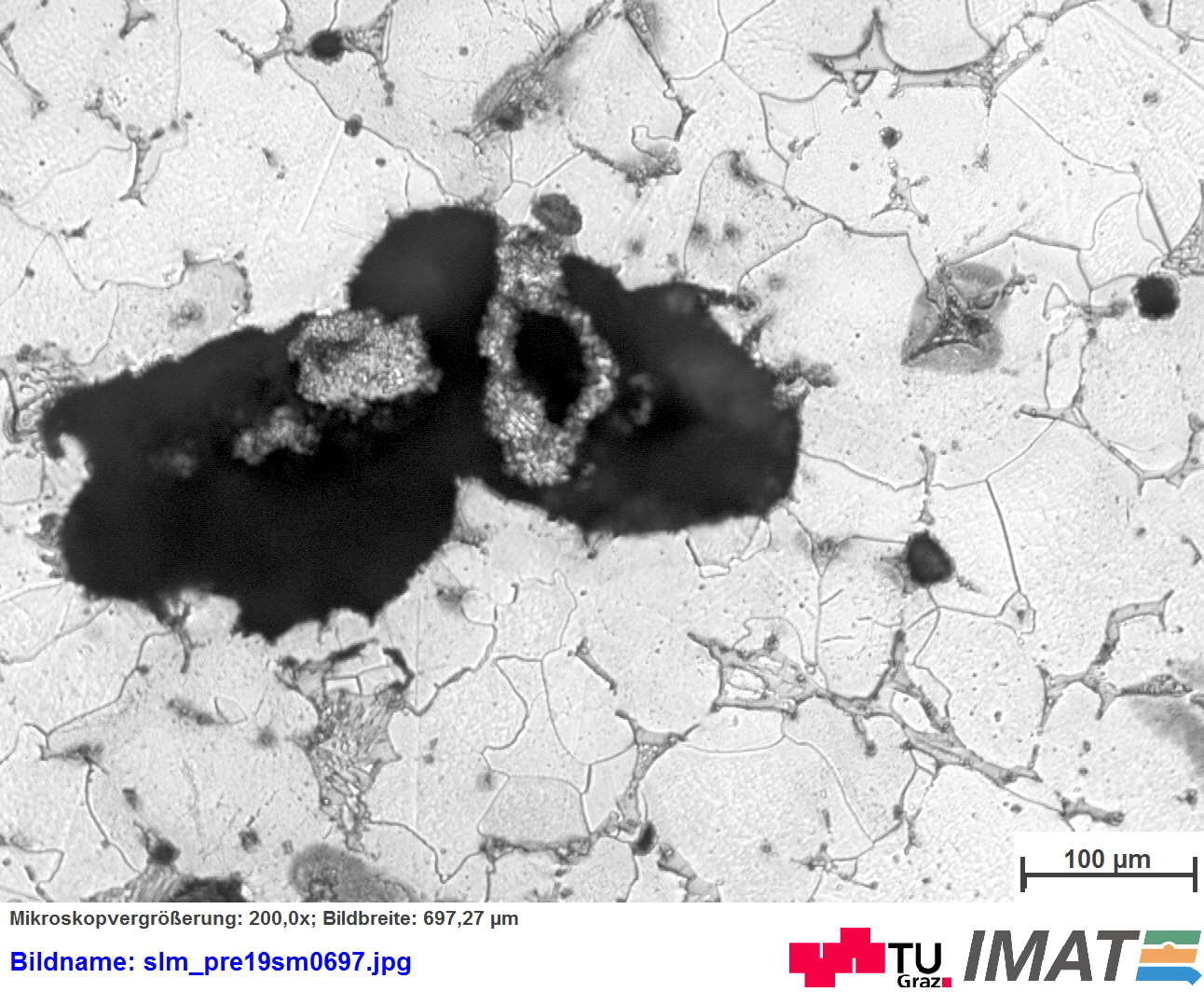

Supplement: Supplementary file 1 [file materials-12-04188-s001.zip › materials-566283-supplementary/Final supplementary/Microstrucutres/B140/slm_pre19sm0697.jpg]

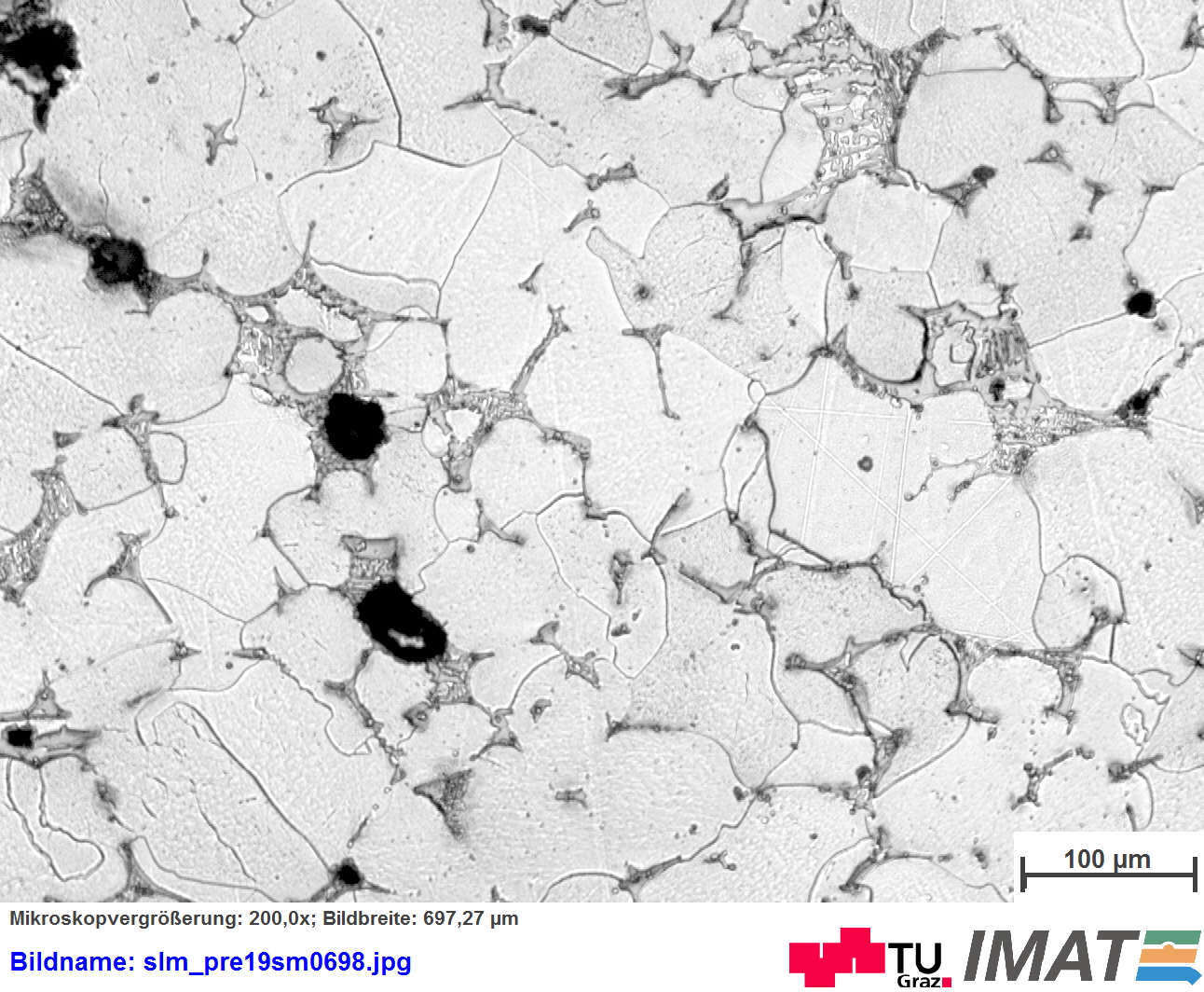

Supplement: Supplementary file 1 [file materials-12-04188-s001.zip › materials-566283-supplementary/Final supplementary/Microstrucutres/B140/slm_pre19sm0698.jpg]

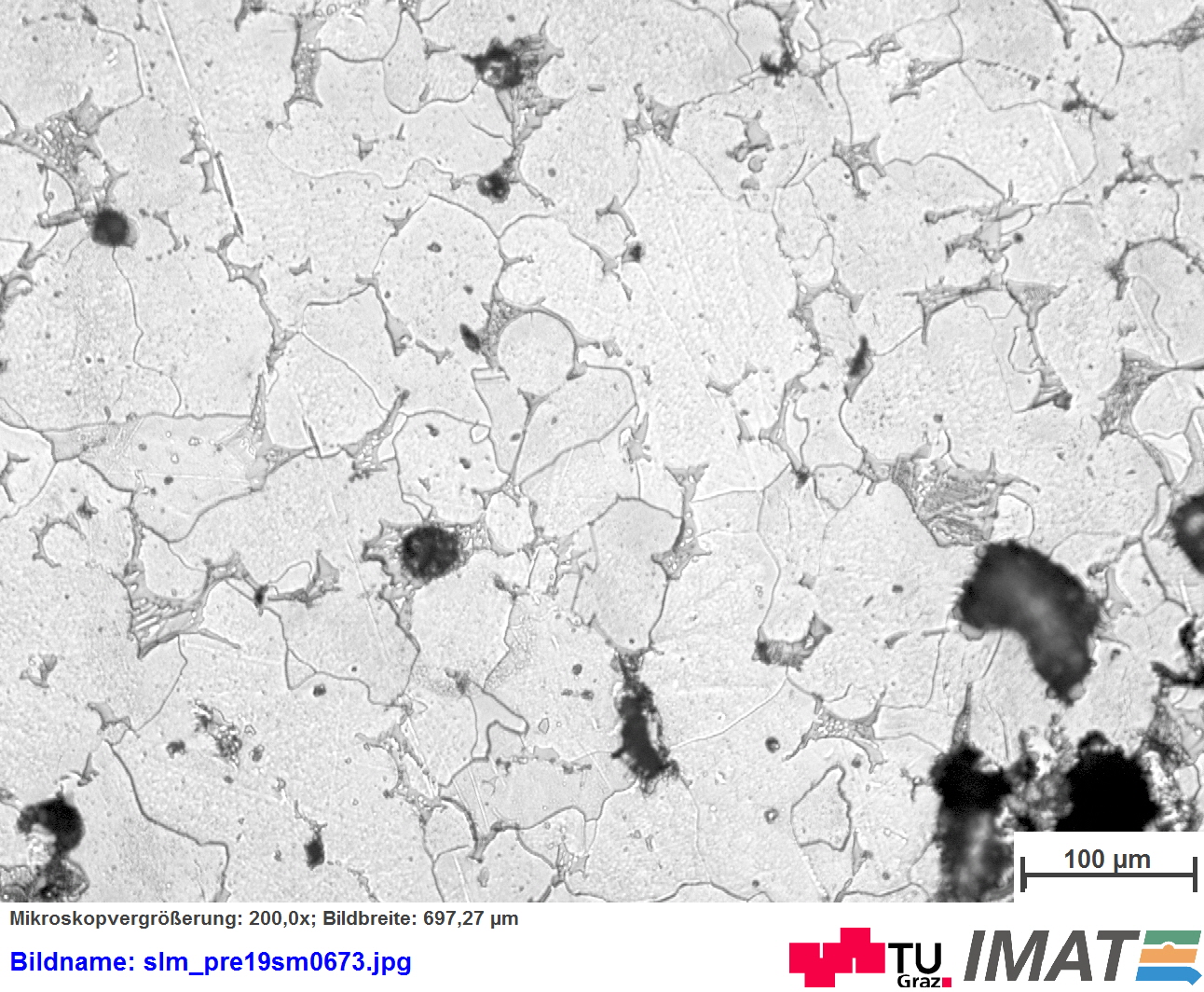

Supplement: Supplementary file 1 [file materials-12-04188-s001.zip › materials-566283-supplementary/Final supplementary/Microstrucutres/B63/slm_pre19sm0673.jpg]

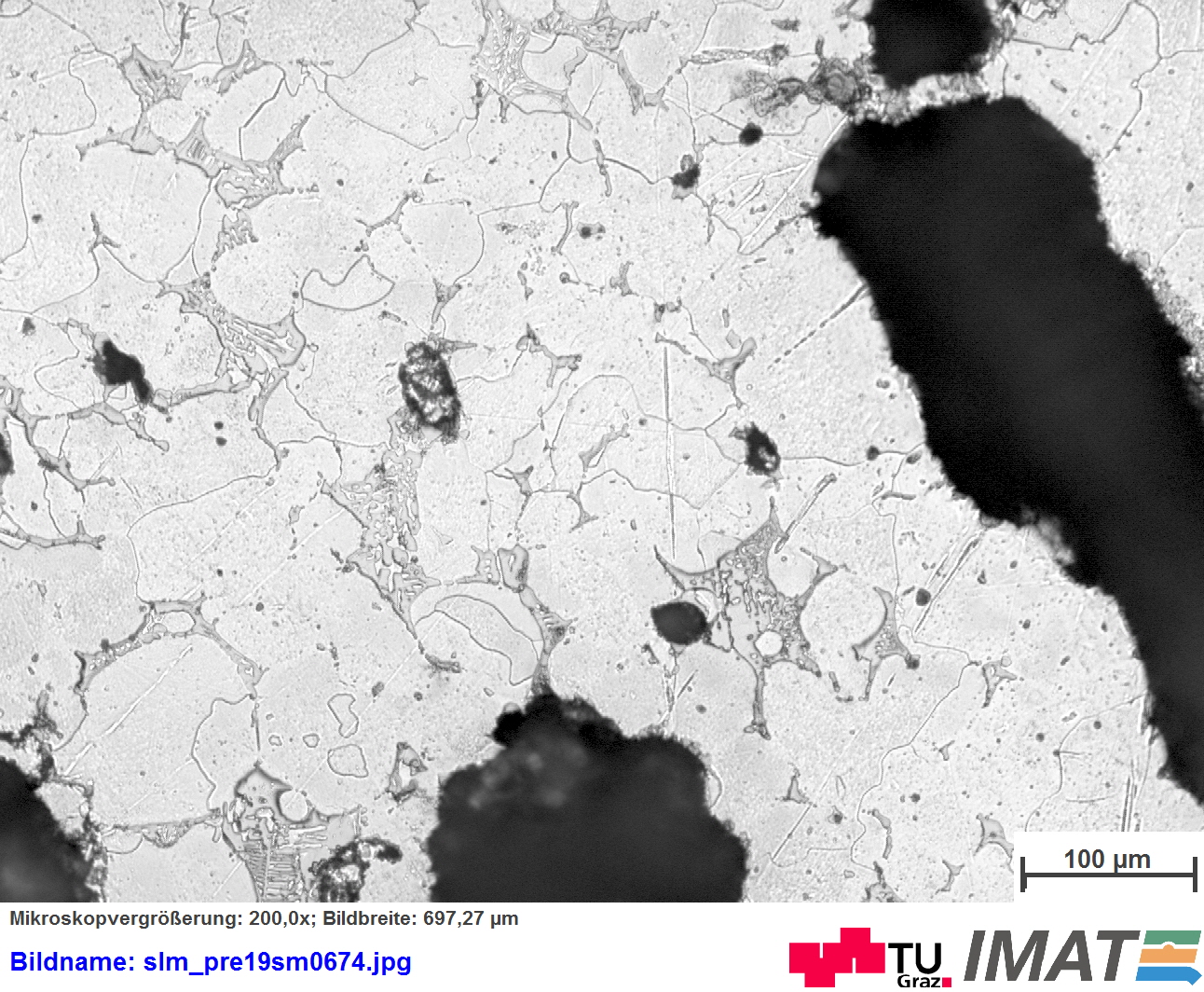

Supplement: Supplementary file 1 [file materials-12-04188-s001.zip › materials-566283-supplementary/Final supplementary/Microstrucutres/B63/slm_pre19sm0674.jpg]

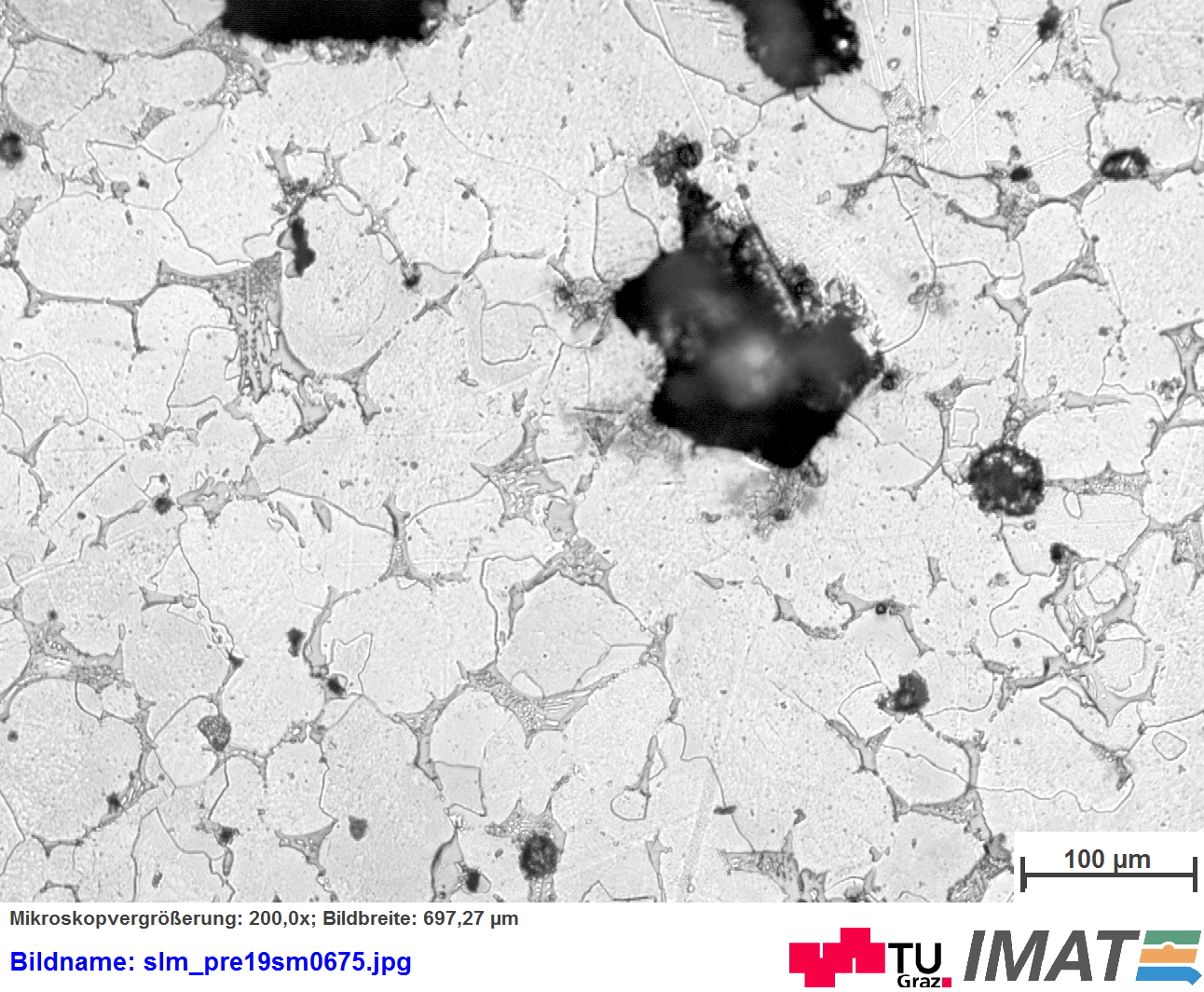

Supplement: Supplementary file 1 [file materials-12-04188-s001.zip › materials-566283-supplementary/Final supplementary/Microstrucutres/B63/slm_pre19sm0675.jpg]

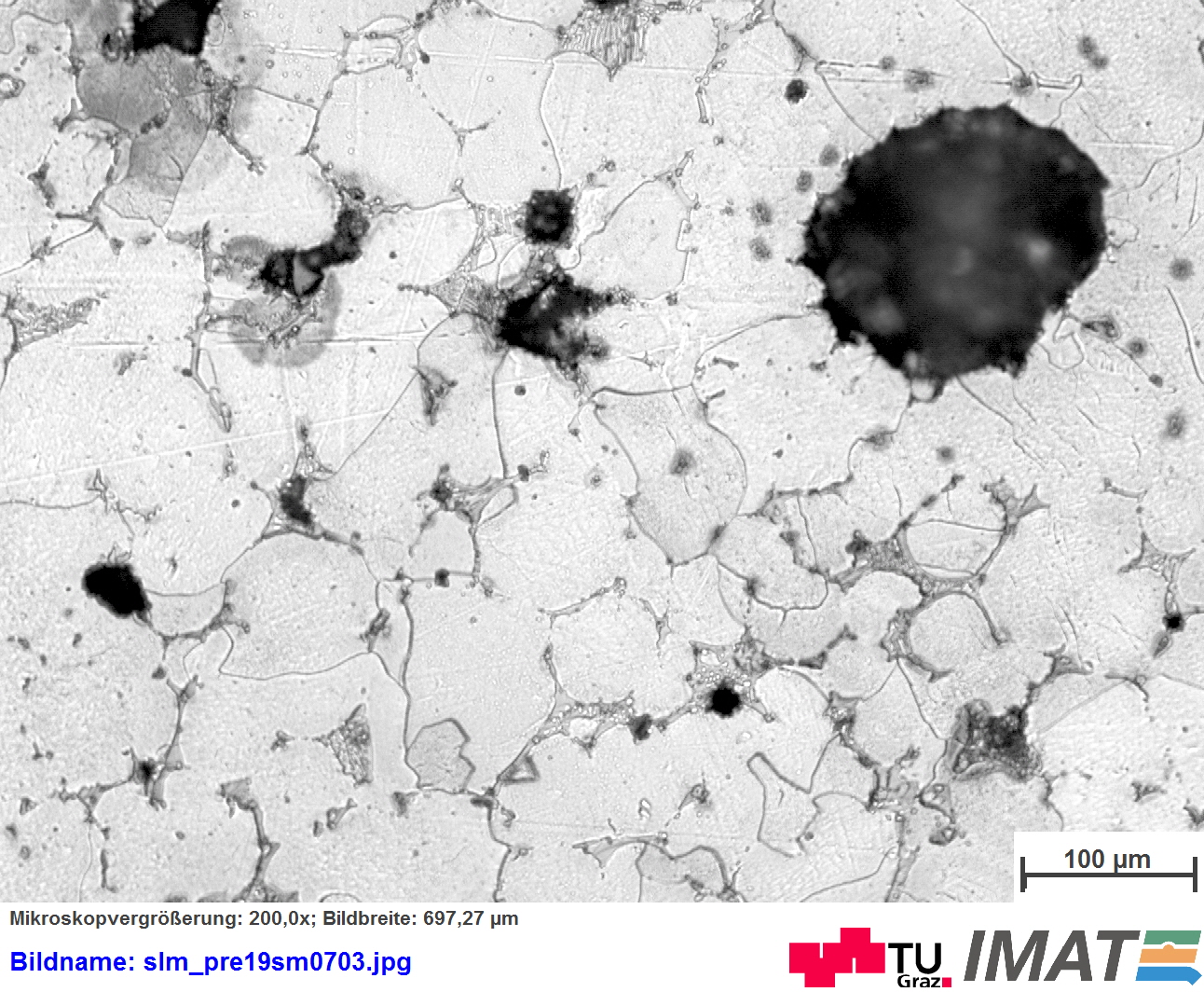

Supplement: Supplementary file 1 [file materials-12-04188-s001.zip › materials-566283-supplementary/Final supplementary/Microstrucutres/C140/slm_pre19sm0703.jpg]

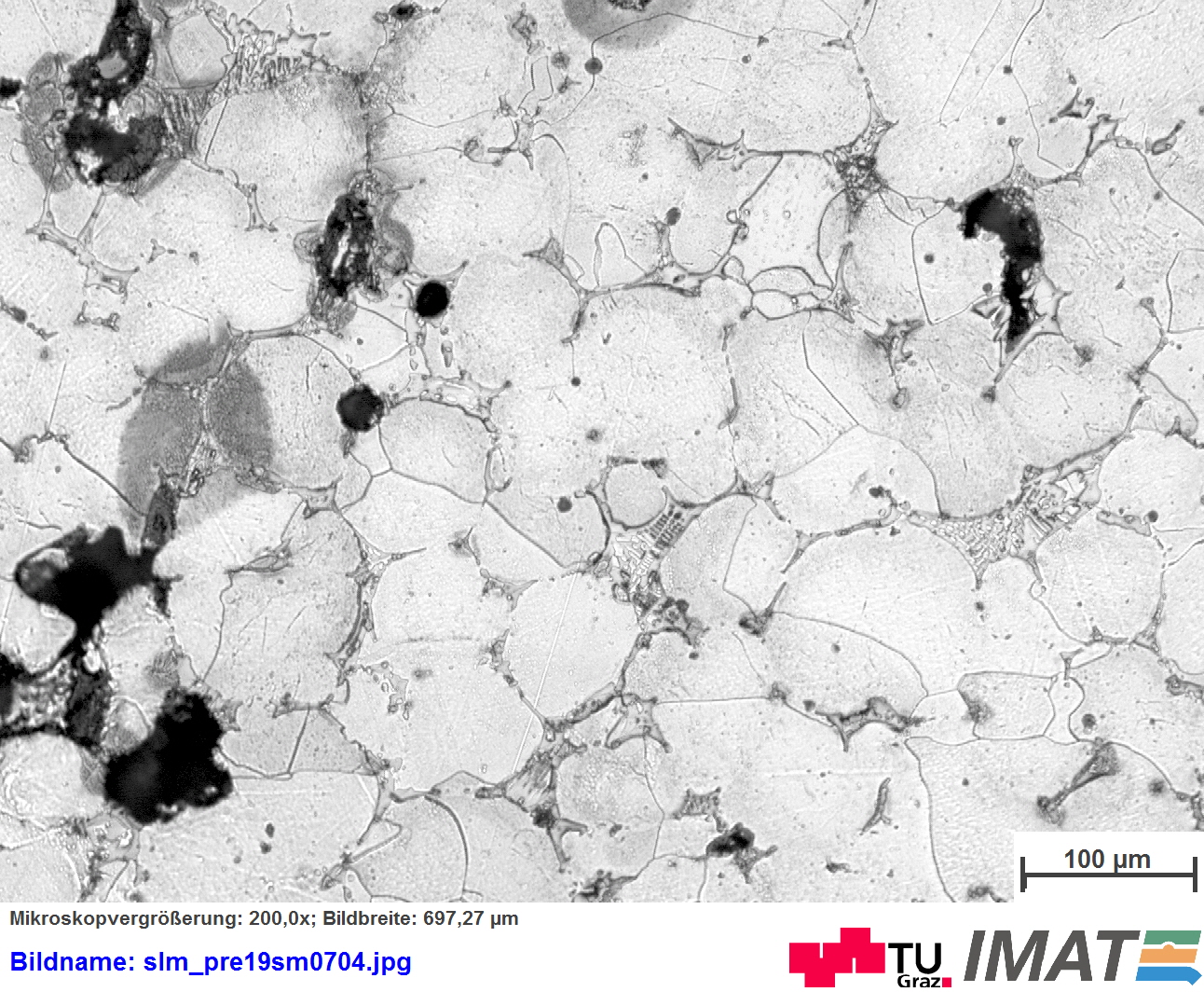

Supplement: Supplementary file 1 [file materials-12-04188-s001.zip › materials-566283-supplementary/Final supplementary/Microstrucutres/C140/slm_pre19sm0704.jpg]

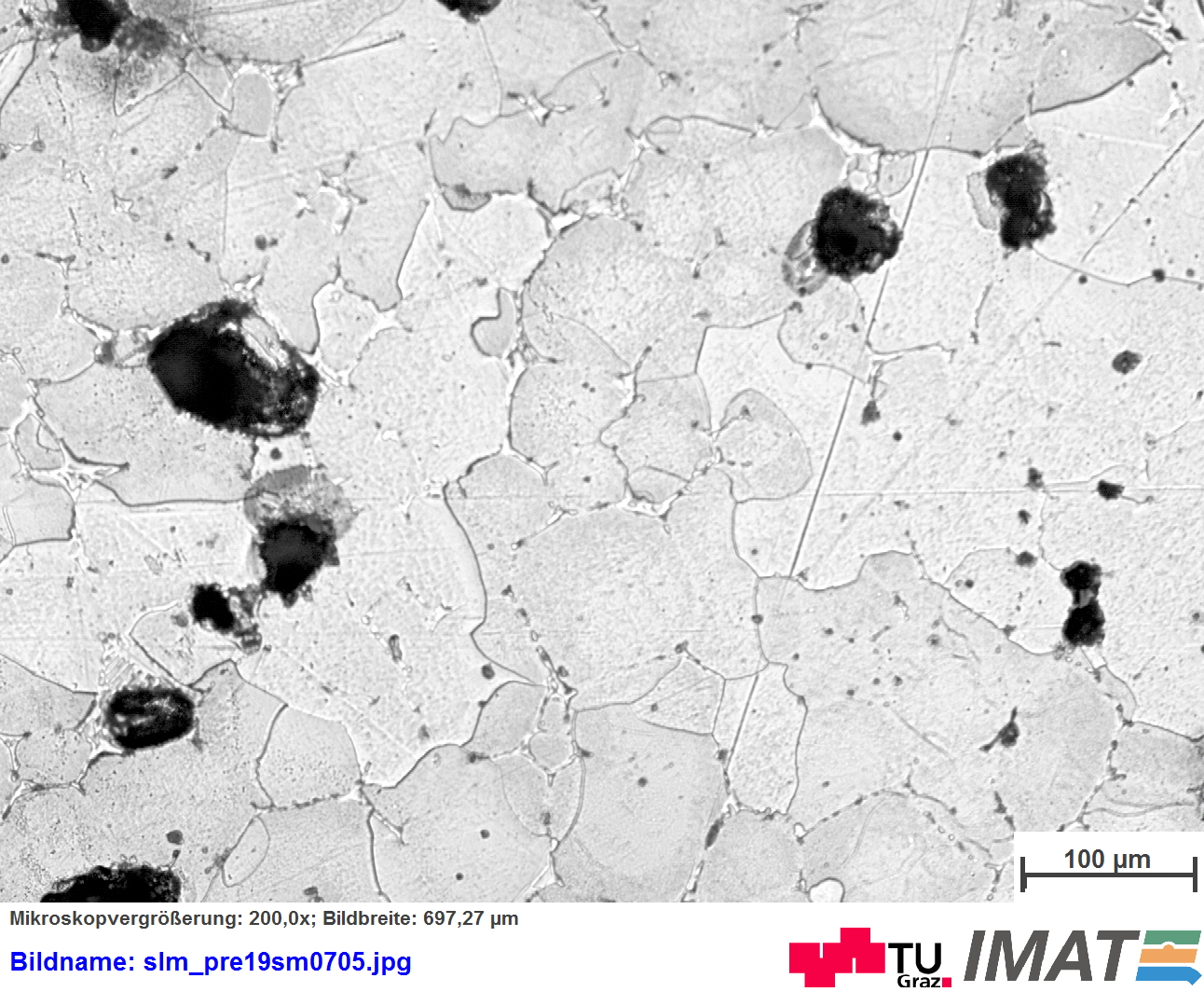

Supplement: Supplementary file 1 [file materials-12-04188-s001.zip › materials-566283-supplementary/Final supplementary/Microstrucutres/C140/slm_pre19sm0705.jpg]

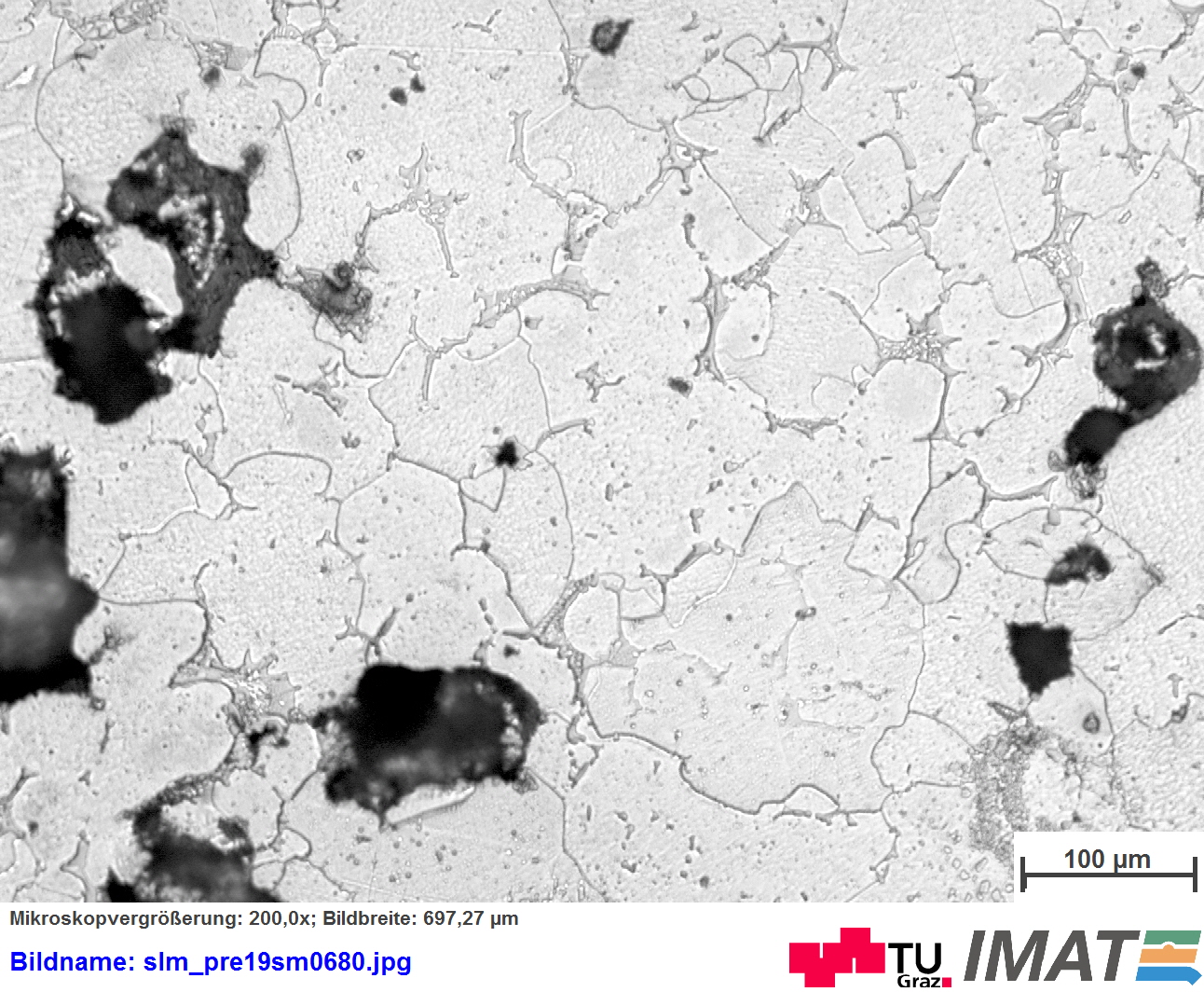

Supplement: Supplementary file 1 [file materials-12-04188-s001.zip › materials-566283-supplementary/Final supplementary/Microstrucutres/C63/slm_pre19sm0680.jpg]

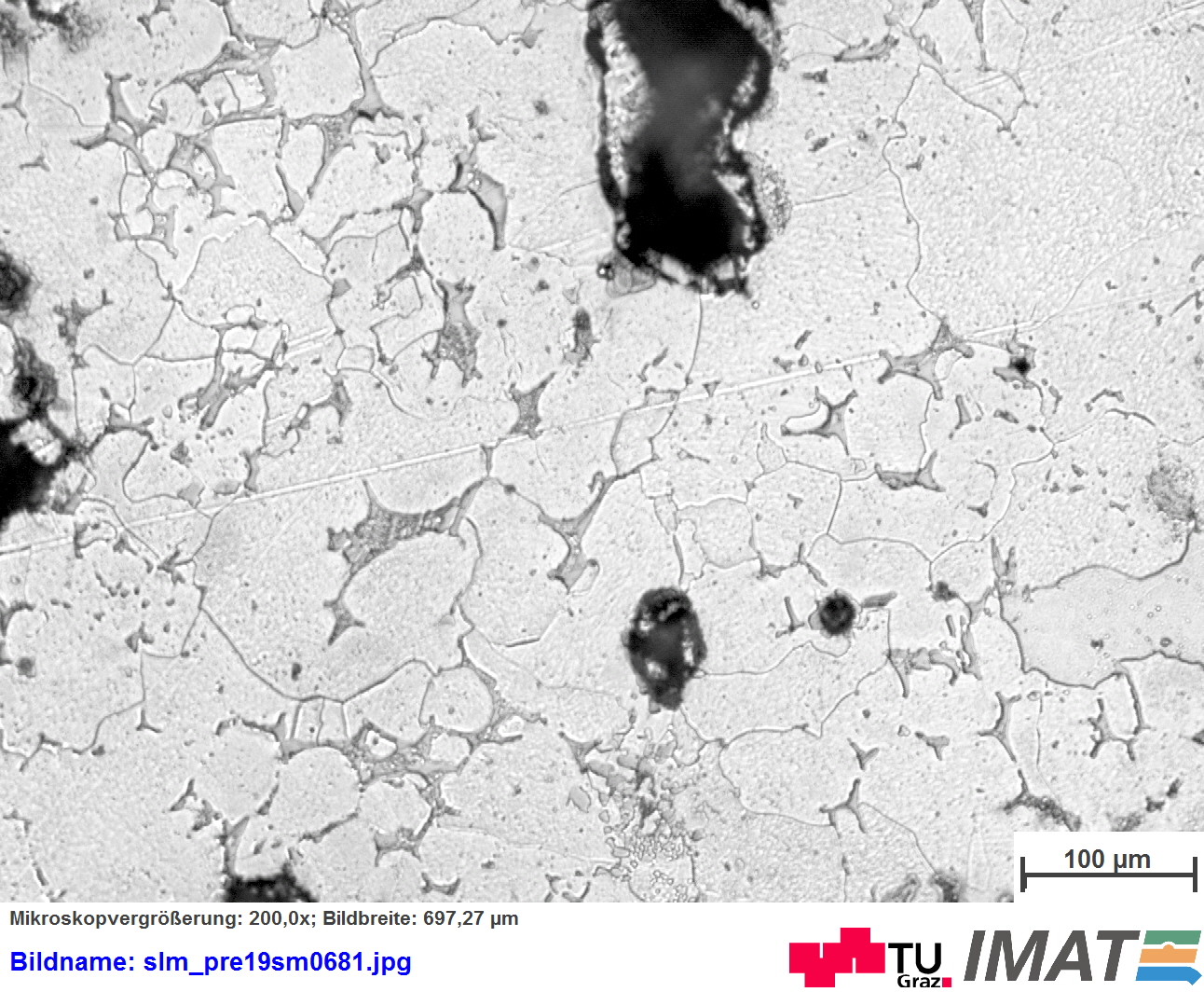

Supplement: Supplementary file 1 [file materials-12-04188-s001.zip › materials-566283-supplementary/Final supplementary/Microstrucutres/C63/slm_pre19sm0681.jpg]

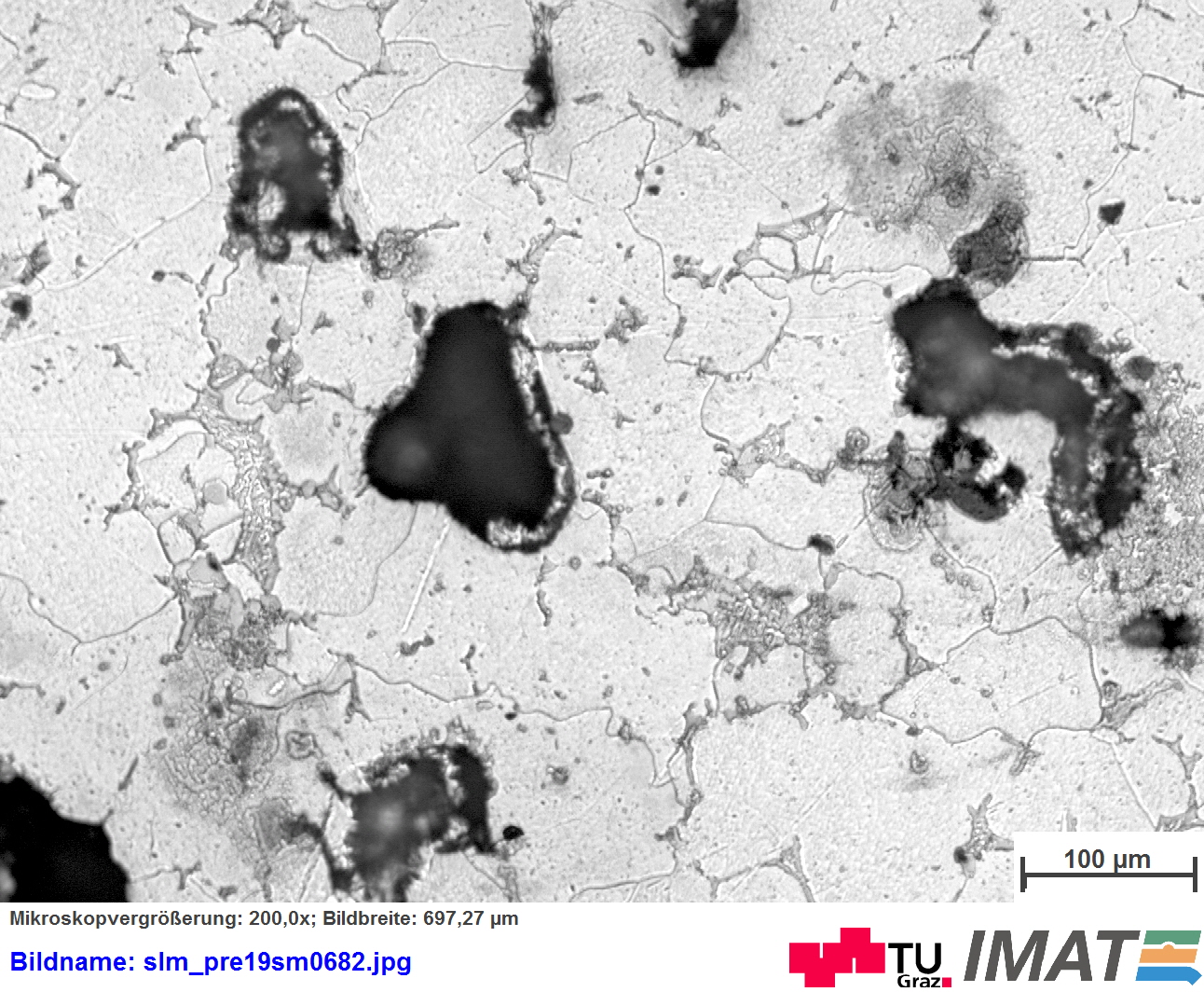

Supplement: Supplementary file 1 [file materials-12-04188-s001.zip › materials-566283-supplementary/Final supplementary/Microstrucutres/C63/slm_pre19sm0682.jpg]

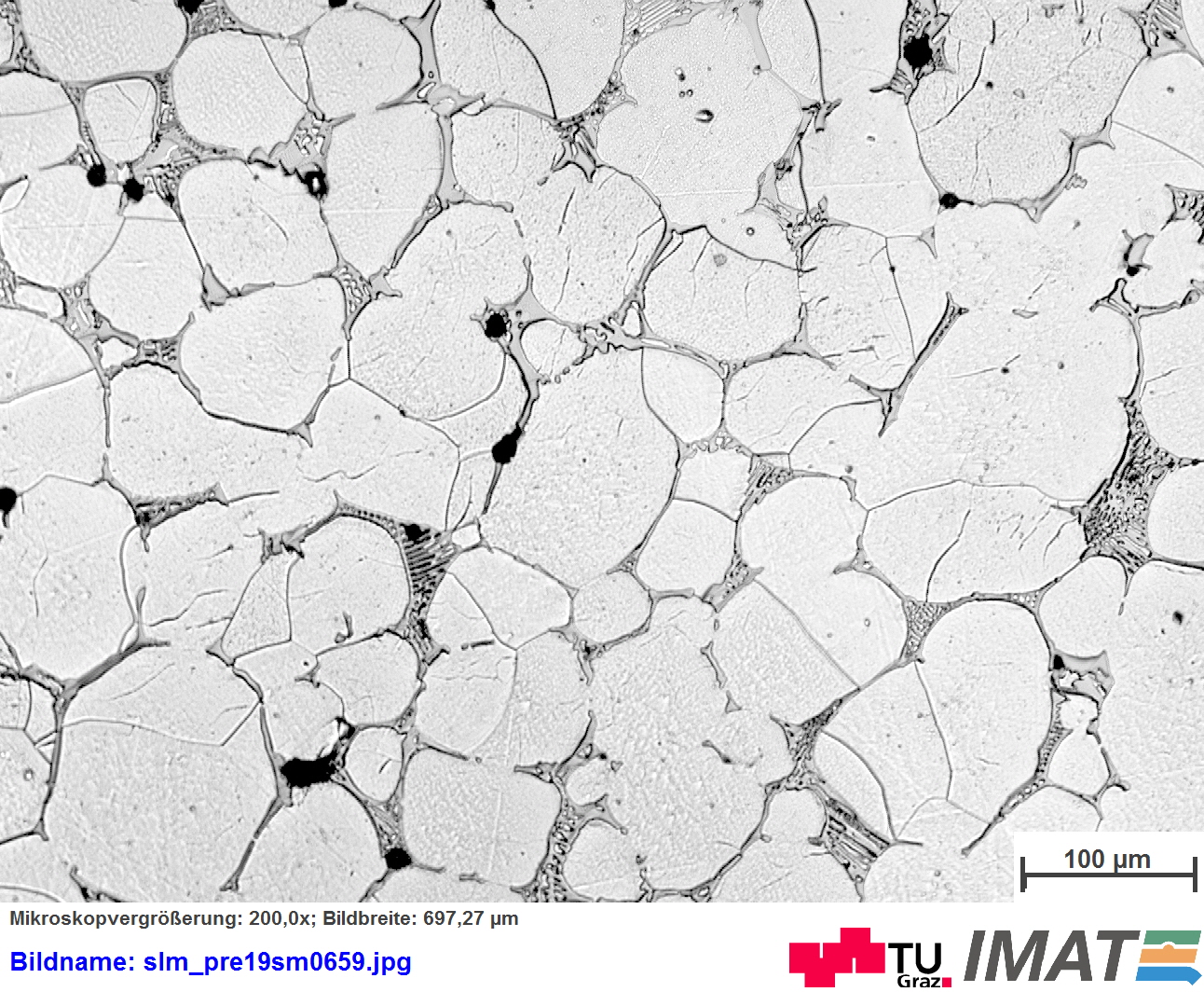

Supplement: Supplementary file 1 [file materials-12-04188-s001.zip › materials-566283-supplementary/Final supplementary/Microstrucutres/REF/slm_pre19sm0659.jpg]

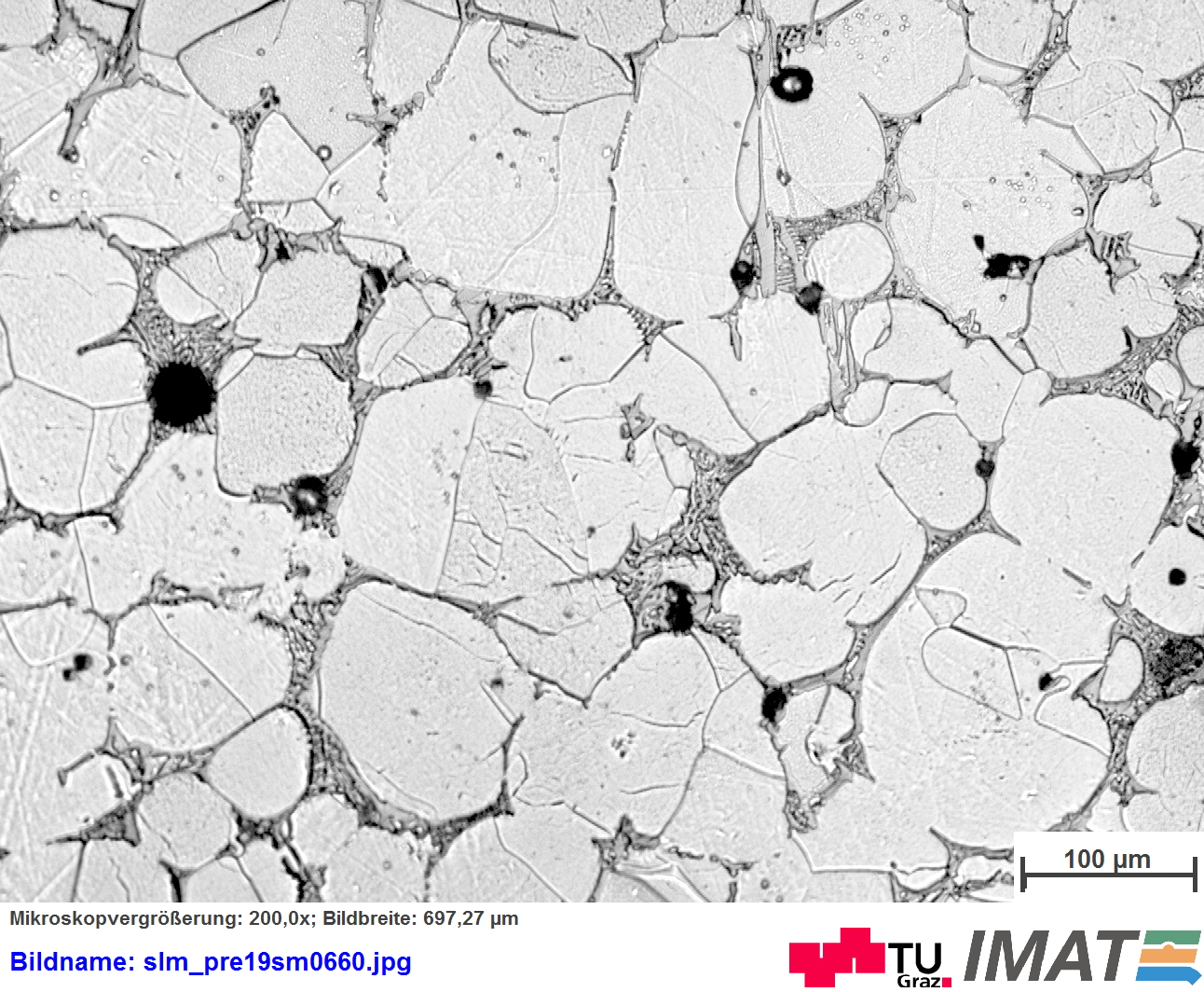

Supplement: Supplementary file 1 [file materials-12-04188-s001.zip › materials-566283-supplementary/Final supplementary/Microstrucutres/REF/slm_pre19sm0660.jpg]

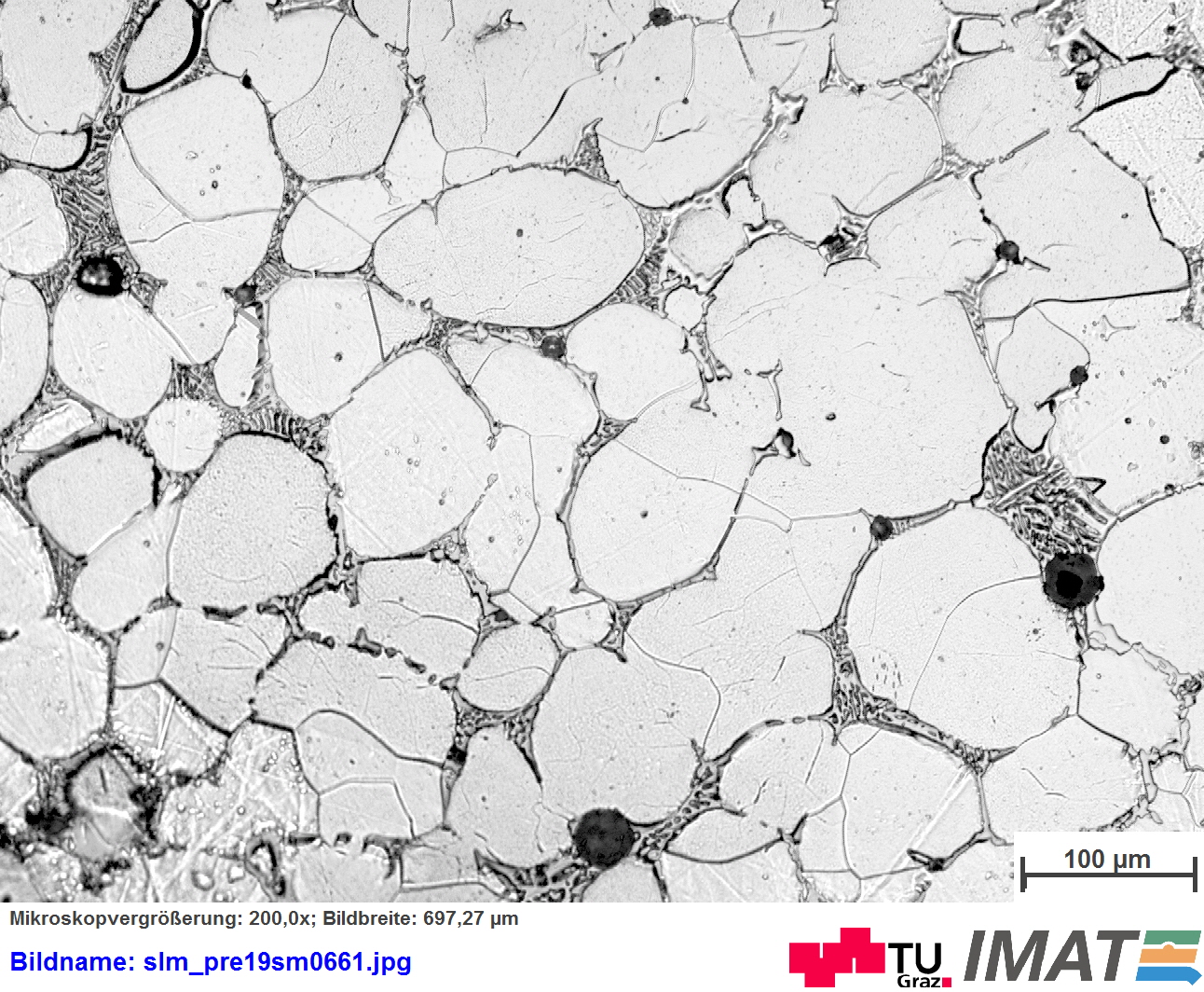

Supplement: Supplementary file 1 [file materials-12-04188-s001.zip › materials-566283-supplementary/Final supplementary/Microstrucutres/REF/slm_pre19sm0661.jpg]
